# Strain and contact-dependent metabolomic reprogramming reveals distinct interaction strategies between *Laccaria bicolor* and *Trichoderma*

**DOI:** 10.1186/s40694-025-00204-w

**Published:** 2025-07-22

**Authors:** Prasath Balaji Sivaprakasam Padmanaban, Pia Stange, Baris Weber, Andrea Ghirardo, Karin Pritsch, Tanja Karl, J. Philipp Benz, Maaria Rosenkranz, Jörg-Peter Schnitzler

**Affiliations:** 1 Research Unit Environmental Simulation (EUS), Helmholtz Munich, Neuherberg, Germany; 2 Professorship Fungal Biotechnology in Wood Science, Wood Research Munich, TUM School of Life Sciences, Technical University of Munich, Freising, Germany; 3 Institute of Plant Sciences, Ecology and Conservation Biology, University of Regensburg, Regensburg, Germany

**Keywords:** Metabolomics, Volatile organic compounds (VOCs), Fungal communication, Mycorrhiza, Bio-control agent, Hyphal composition

## Abstract

The genus *Trichoderma* (Hypocreaceae, Ascomycota) compromises over 400 known species, that are found in various soils, on plant surfaces and as plant endophytes. Interactions between the mycoparasitic *Trichoderma spp.* and beneficial ectomycorrhizal fungi such as *Laccaria bicolor* (Hydnangiaceae, Basidiomycota) can influence the structure of fungal communities and plant symbioses. In this study, we conducted in vitro dual-culture experiments involving *L. bicolor* and four *Trichoderma* strains *(T. harzianum WM24a1, MS8a1, ES8g1, and T. atrobrunneum)* to analyze their metabolic responses in relation to varying degrees of physical contact. Using integrated analyses of volatile organic compounds (VOCs), hyphal metabolomes, and secreted exudates, we uncovered strong contact- and strain-dependent growth inhibition patterns: *Trichoderma* growth was suppressed under shared headspace, whereas *L. bicolor* was more strongly inhibited under direct contact. Metabolomic profiling revealed distinct and strain-specific alterations in both VOC and soluble metabolite profiles during co-cultivation, with hundreds of discriminant mass features affected. Key metabolic pathways, including amino acid, carbohydrate, lipid, and secondary metabolite biosynthesis, showed differential enrichment depending on the interaction stage and fungal partner. These results demonstrate that *Trichoderma–Laccaria* interactions are mediated by dynamic, contact-specific chemical reprogramming and suggest that fungal recognition and competition involve coordinated changes in both volatile and non-volatile metabolite production. Our findings provide a foundation for exploring how such antagonistic interactions may influence tripartite communication in plant-associated microbial networks. They also highlight the potential role of both emitted and secreted fungal metabolites in shaping interaction dynamics through putative non-self-recognition mechanisms.

**Supplementary Information:**

The online version contains supplementary material available at 10.1186/s40694-025-00204-w.

## Background

The rhizosphere is home to a diverse range of microbial species, including bacteria, yeasts, protozoa, archaea and fungi, all of which play an important part in soil health and plant well-being [1, 2]. When microbial bioinoculants are used as biocontrol agents, competitiveness and proper establishment in the rhizosphere are prerequisites for effective biocontrol regardless of the mode of action of the biocontrol agents (BCA) used [3–7]. Among the available BCAs, fungi play multiple roles with high plasticity and adaptability to adverse and unfavourable conditions [8]. Currently, *Trichoderma* spp. dominate the bioinoculant market with many of the 400 identified species showing biocontrol activities [9–11]. *Trichoderma* spp. occur in diverse environments [12] and in complex interactions with other soil microflora [8]. They can control and antagonise plant pathogenic fungi through several direct and indirect mechanisms [13], including competition for space and nutrients [14, 15], secretion of various hydrolytic enzymes such as chitinases, glucanases and proteases, or emission of volatile organic compounds (VOCs) [10, 16, 17]. Some effects of fungal antibiotics and effector molecules have also been reported in fungal confrontations [18–20]. *Trichoderma* spp. is especially interesting BCA, as it does not only antagonize pathogens, but it also may have the potential to discriminate between plant-beneficial and pathogenic fungi [16, 21]. Recently, [16] showed that the growth of *Trichoderma* spp. display positive tropism towards plant pathogens and a negative tropism in the presence of ectomycorrhizal (ECM) fungi, such as *Laccaria bicolor*. In this interaction, the possible ways in which the communication could occur is either via VOCs, which have been previously shown to play a role in fungal-plant and fungal-fungal interactions [10, 22, 23] or via soluble metabolites. These compounds could be important players in the mutual perception of the studied fungi.

The term VOCs encompasses a wide range of chemically diverse small molecules released by plants, microbes and fungi [10, 24, 25]. To date, approximately 480 different VOCs have been detected from *Trichoderma* spp. [26, 27], whereas only 15 VOCs, mostly terpenes, have been reported from *L. bicolor* [10, 22, 28]. *Trichoderma* VOCs include heterocycles, aldehydes, ketones, alcohols, phenols, thioalcohols, thioesters and their derivatives and hydrocarbons such as monoterpenes and sesquiterpenes and their derivatives, i.e., oxygenated monoterpenes and oxygenated sesquiterpenes [26, 27]. Previous studies on *Trichoderma* spp. VOC production have shown that their emission profiles vary depending on the species or strains and on the cultivation environment [29, 30]. The VOC profiles depended, moreover, on the contact degree with other ECM fungi (*L. bicolor*), i.e. the emission profiles were altered depending on if the communication occurred only through headspace (Aerial Contact-AC), shared growth media (Media Contact-MC) or direct hyphal contact (Direct Contact-DC) [10]. However, the basis of interactions between *Trichoderma* spp. added to soils and different fungi (e.g. ECM) present in the rhizosphere remains unclear.

In addition to VOCs, the confrontation between different fungal hyphae leads to changes in mycelial growth, composition and secretion of soluble secondary metabolites [31–33]. Such bioactive allelochemicals are “non-nutritional chemicals produced by individuals of one species that affect the growth, health, behaviour, or population biology of another species” [34]. The genomes of *Trichoderma* spp. and *L. bicolor* are rich in genes encoding enzymes responsible to produce secondary metabolites [35–37]. These metabolites, which include, among others, signalling molecules, growth inhibitors, toxins and their by-products [32, 38, 39], can contribute to a potential competitive advantage for *Trichoderma*’s biocontrol activity. Soluble metabolites may also mediate various fungal-plant interactions, for example in establishing a symbiotic relationship between *L. bicolor* and the host [35, 40]. Understanding the recognition and interaction between *L. bicolor* and *Trichoderma* spp. is crucial given their co-occurrence in shared niches and rhizospheres, such as that of poplar [10, 16, 41, 42] and the common use of *Trichoderma* spp. as BCA. As for BCA, the extent of the interaction effects depends on the duration of inoculation and the concentration of secreted allelochemicals [43]. Only in the recent years, there has been an increased focus on deciphering the chemical composition of allelochemicals released by *Trichoderma* and their effects on biochemical and physiological processes, with potential field applications [44]. Further studies are essential to understand the function of individual metabolites in the interaction of *Trichoderma* spp. with other, potentially beneficial fungi sharing the same habitat.

While our previous work focused on VOC-mediated signalling in *Trichoderma–Laccaria* interactions [10], the present study extends this framework by incorporating hyphal and exudate metabolomics. By combining volatile profiling with non-volatile intracellular and extracellular metabolite data, we provide a more comprehensive view of the chemical landscape underlying fungal interactions. This multi-layered approach allows us to dissect not only airborne communication, but also contact-mediated and diffusible metabolite exchanges. This reveals distinct spatial and temporal signatures of fungal responses. Such an integration offers new insights into the complexity of fungal non-self-perception and interaction strategies which cannot be captured through VOC analysis alone. Therefore, in the present study, we confronted the ectomycorrhizal fungus *L. bicolor* with either of four mycoparasitic *Trichoderma* spp.—*T. harzianum* strains WM24a1, MS8a1 and ES8g1 and *T. atrobrunneum* [10, 16, 45]. As *Trichoderma* and *Laccaria* are known to be naturally associated with deciduous tree species such as *Populus* spp. [36, 46], we aimed to track changes in the dynamics of volatile and soluble metabolites to elucidate the interaction between *Trichoderma* spp. and *L. bicolor* over distance and time. We hypothesize that (I) interaction-driven alterations in volatile and soluble metabolite profiles will manifest in a manner dependent on the level of physical contact and the fungal strain and (II) these metabolite changes represent distinct signalling or antagonistic strategies employed by each fungal partner.

## Methods

### Fungal strains, media and cultivation

Three strains of *Trichoderma harzianum* (WM24a1, MS8a1 and ES8g1), *Trichoderma atrobrunneum* and *Laccaria bicolor* (strain S238N) were used in the study. The strains were selected based on preliminary data from an in vivo plant-interaction study [10] and from related studies [16, 23, 47]. Prior to co-cultivation, the fungi were precultured on a “Modified Melin-Norkrans (MMN)” synthetic medium (Supplementary Method S1) as described by Müller and colleagues [28], at room temperature in darkness.

The fungi were inoculated as mycelial plugs (1 cm diameter) in glass Petri dishes of 10 cm diameter containing 40 ml of Modified Melin-Norkrans (MMN) synthetic medium covered with a sterile cellophane sheet for the main experiments (as previously described [28]) in five replicates for each condition. The inoculated plates were sealed with parafilm and incubated in permanent darkness at 23 °C in a growth chamber.

### Validation of *Trichoderma* species and strain identities

The validation of the species and strain identity of the *Trichoderma* isolates *T. harzianum* (WM24a1) and *T. atrobrunneum* was carried out by Stange et al. [47] following Cai and Druzhinina [11], and the reports were added to the supplementary file sets in OSF. The other *Trichoderma* isolates (*T. harzianum* (MS8a1 and ES8g1) were obtained from the collection of the Austrian Institute of Technology (AIT), and identification of the strains was carried out following the previously published guidelines [11]. Since ITS markers can only identify *Trichoderma* at the genus level, two additional phylogenetic markers -translation elongation factor 1α (tef1) and RNA polymerase II subunit B (rpb2) were amplified for further analysis using the previously described primers [47] (Supplementary Method S2). The tef1 and rpb2 sequences were trimmed using TrichoMark 2020 and then aligned for multiple sequence comparison through the MUSCLE algorithm [48] based on log expectation. A phylogram was generated using tef1 and rpb2 sequences and analysing them with the maximum-likelihood (ML) method in the IQ-TREE web server [49, 50], using 1000 bootstrap replicates and the best-fit model TIM2e + G4 [51]. The resulting phylogenetic trees were visualised with the Interactive Tree of Life (iTOL, version 5) [52]. The corresponding ITS, tef2 and rpb sequences were included in supplementary file sets. The available ITS, tef and rpb2 data of other *Trichoderma* strains from Cai and Druzhinina [11] were included in the phylogenetic tree to confirm the species identity. Since the rpb2 sequence was incomplete for the used MS8a1 strain and considering the complexity of the *T. harzianum* clade [53, 54], the strain was reported as available in the nomenclature of AIT collection as *T. harzianum* (MS8a1). The respective phylogenetic tree can be found in the Supplementary Figures (Fig. S1 A, B). The used *Laccaria bicolor* S238N strain was obtained from the collection of the Institute National de la Recherche Agronomique (INRA), Nancy, France.

### Experimental setup and growth analysis of fungi

*Laccaria bicolor,* as a slow-growing species, was initially inoculated on one side of the Petri dishes for co-culture. Split Petri dishes were used to study the interactions in AC and unsplit Petri dishes for other interactions (MC and DC). After 14 days of cultivation, (according to the growth stages defined previously [10]), an agar plug of the *Trichoderma* strain was inoculated on the other side of the same Petri dish. Five replicates of each *Trichoderma* strain in each interaction scenario and pure cultures (PC) were prepared and examined according to Guo and colleagues [10]. Images were taken at 3,5 and 7 days after co-culture using a Nikon D300 camera (60 mm Nikkor AF-S Micro-Nikkor Lens, Nikon, Tokyo, Japan) and used for growth analysis. The pure cultures (Supplementary Fig. S2) were also grown under similar conditions and analysed on the same timeline as with the co-cultures. Growth inhibition was calculated using the following formula [55]:

$${\text{Growth}}\,{\text{Inhibition}}\,\left( \% \right) \, = {\text{ D1}} - {\text{D2}}/{\text{D1}} \times {1}00$$

D1: fungal area grown alone.

D2: fungal area grown in co-cultivations.

Three days post *Trichoderma* inoculation, the headspace VOCs were collected from the five replicates of PC, AC and MC co-cultivation scenarios. Similarly, mycelium and media from PC, MC and DC were used for non-targeted metabolomic analysis. To enable direct comparison of metabolite profiles under identical temporal conditions, pure cultures of each fungus were sampled in parallel at the same time points as the co-cultures. Although sampling was performed at fixed intervals to ensure consistency across treatments, we recognize that differences in fungal growth rates and interaction-induced inhibition may have affected the physiological stage of each species at the time of sampling. To minimize variation, pure cultures were sampled alongside the co-cultures at each corresponding time point.

### VOC profiling

VOCs from the five replicates of each condition were extracted from the headspace of PC, AC and DC fungal co-cultures for 16 h using Twisters (Gerstel GmbH & Co.KG, Mülheim an der Ruhr, Germany) and employing Headspace Sorptive Extraction (HSSE) technique according to the previously described method [28]. Subsequently, the Twisters were stored at 4 °C and later subjected to VOC analysis through Thermal Desorption-Gas Chromatography-Mass Spectrometry (TD-GC–MS), adhering to protocols as previously detailed [23, 56, 57].

Putative annotation of the mass spectra was performed by comparison with best hits from the libraries of reference spectra (NIST 11, Wiley 275). For quantification, response factors were calculated using standards: sabinene and α-pinene for monoterpenes, linalool for oxygenated monoterpenes, β-caryophyllene and α-humulene for sesquiterpenes and geraniol for oxygenated sesquiterpenes. The resulting data were input for subsequent statistical analysis (in supplementary files).

### Non-targeted metabolomic analysis

The area of mycelium and media (beneath the mycelium and cellophane) of MC, DC and PC was divided into three zones (Figs. 4A and 6B). The mycelium was scraped from the cellophane with a spatula; the cellophane was removed after sampling the hyphae and the media was sampled from the same area where the mycelium was collected from 5 replicates of each experimental conditions. The collected samples were stored at − 80 °C for further processing and extraction. The media were freeze-dried at − 50 °C under 0.040 mbar vacuum (Alpha 1–4 LDplus, Christ, Osterrode, Germany). The extraction process followed the protocol described by Bertić et al. [58] (Supplementary Method S3). A preliminary experiment was performed with hypha/media extracts from freeze-dried and non-freeze-dried samples to select the appropriate LC–MS chromatographic conditions. Chromatographic runs were performed on RP18 and HILIC columns in both positive and negative electrospray ionisation (ESI) modes. To detect both polar and non-polar metabolites and mass features, one ionization mode was used for each column (HILIC + mode and RP—mode). This ensured that most mass features (> 75%) detected in the samples were covered.

Untargeted metabolomics analysis was carried out following the methodologies outlined by Ghirardo et al. [59] and Hemmler et al. [60] utilizing an ultra-performance liquid chromatography (UPLC) system coupled with ultra-high resolution (UHR) tandem quadrupole/time-of-flight (QqToF) mass spectrometry (MS). The instrumentation comprises an Ultimate 3000RS UPLC system (Thermo Fisher, Bremen, Germany), a Bruker Impact II QqToF mass spectrometer, and an Apollo II electrospray ionisation (ESI) source (Bruker Daltonic, Bremen, Germany), Each sample underwent separate analysis using the RPLC-positive (+) and HILIC-negative (-) columns of ESI modes. Detailed parameters are described in Supplementary Methods S3.

### Metabolic profiling

The LC–MS data were analysed using Metaboscape 4.0 (Bruker^®^). This software facilitated post-acquisition processes, including peak picking, alignment, isotope filtering and peak grouping based on peak-area correlations (as in [61]). Detailed parameter settings are provided in Supplementary Methods S4 & S5. Results from RP and HILIC analyses were merged manually.

Putative annotations were based on available MS/MS spectra matched to different libraries, including HMDB (http://www.hmdb.ca/) [62], MoNa and Vaniya/Fiehn Natural Products Library using the LC–MS/MS spectra. For the other features the smart formulas (chemical formula) classification based on the elemental ratios and mass (m/z) was used for putative annotation. The chemical formulas were further confirmed using the annotation by in-house built R script and metabolite database [58]. However, only smart formulas from this putative annotation were used to avoid misinterpreting the actual compound. The chemical class classification of the significant features was performed using the'Multidimensional Stoichiometric Compound Classification'(MSCC) approach, based on elemental ratio using the composition of C, N, H, O, P, S, O:C, N:C, H:C, P:C and N:P ratios [63].

The data were pre-processed to remove blanks from the LC column, normalised using the peak intensities of internal standard (IS) mixture and missing values were replaced with the random values below the detection limit (1–800). For the metabolic data from the blank media, in addition to the standard processing used for the hyphae data, the mass features present in the blank media were subtracted from samples of plates with fungi to avoid misinterpretation of the media components and used for comparison. The resulting data were used for subsequent statistical analysis (in supplementary files).

### Statistical analysis

The growth area of the fungal mycelium from the five replicates was measured using the image analysis software IMAGEJ [64]. The significance of growth inhibition was tested by a one-way ANOVA followed by Tukey HSD post hoc test (p < 0.05). All data were always logarithmically (log10) transformed, centred and Pareto scaled [58]. Principal component analysis (PCA) and orthogonal partial least square regression discriminant analysis (OPLS-DA) of both VOC and metabolic data were performed in SIMCA-P v.13.0.3.0 (Umetrics, Umea, Sweden). Features with a Variable Importance of Projection (VIP) score greater than 1 were selected for the calculation of log2fold change (> 1), p-value and adjusted p-value (< 0.05) to identify potential discriminative mass features. Detailed processing parameters can be found at [58]. Hierarchical clustering analysis (HCA) of discriminant VOC and metabolic mass features was used to visualise expression patterns. Enrichment- and pathway analysis of the putatively significant annotated features was performed using Metaboanalyst 5.0 (https://www.metaboanalyst.ca/MetaboAnalyst/home.xhtml) [65]. The resulting pathways were cross-checked using the pathway analysis function in Metaboanalyst 5.0 (KEGG). The occurrence of the pathway function in corresponding fungi and the biological process of the pathway were manually checked on the KEGG database (https://www.genome.jp/kegg/pathway.html) [66]. Alluvial plots were generated using RAWGraphs 2.0 [67].

## Results

Given the complexity of analysing four *Trichoderma* strains across multiple interaction types and time points, the focus is on presenting the detailed results on one representative strain, *T. harzianum* MS8a1, which exhibited distinct metabolic profiles and interaction dynamics. Comprehensive results for the remaining strains are provided in the Supplementary Information.

### Co-culture of *L. bicolor *and *Trichoderma* mutually limit mycelial growth

**Fig. 1:**
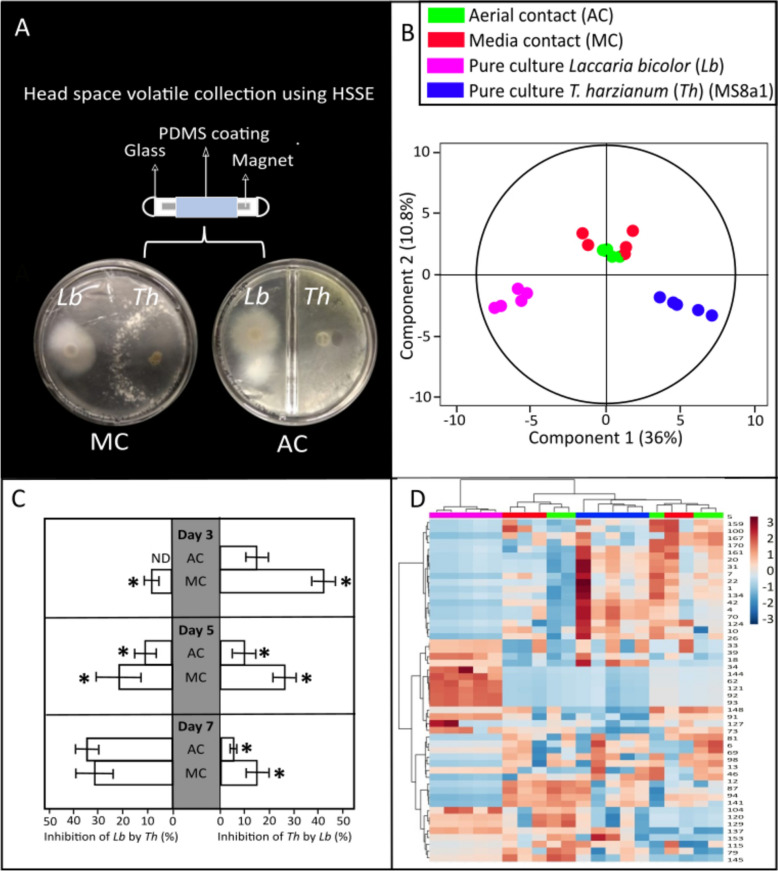
VOC analysis of *L. bicolor* (*Lb*) and *T. harzianum (*MS8a1*)* (*Th*) co-cultivated either in aerial contact (AC) or media contact (MC). **A** Experimental setup of the VOC analysis from the fungal co-cultivation in split and non-split Petri plates. **B** Orthogonal partial least square regression discriminant analysis (OPLS-DA) showing differences among VOC profiles under different levels of co-cultivation over time. OPLS model fitness: R^2^X(cum) = 0.855, R^2^Y(cum) = 0.787, Q^2^Y(cum) = 0.625, CV-ANOVA = 7.2 × 10^–7^. **C** Growth inhibition of *Lb* by *Th* (left) and *Th* by *Lb*(right) under different levels of co-cultivation compared to growth on pure cultures. Significances within each day are denoted as asterisks (one-way ANOVA and Tukey HSD, p < 0.05); Mean ± SE; values are average of 5 replicates. ND: not detected. **D** Two-dimensional hierarchical clustering analysis of the VOC emissions from the two fungi grown as MC, AC or pure culture

When incubating *L. bicolor* with *Trichoderma* spp. in co-culture (Fig. 1A), different growth inhibition dynamics could be observed (Fig. 1C, and Supplementary Figs. S3C, S4C, S5C). Particularly, in the context of strain MS8a1, on the day three, MS8a1 did not inhibit *L. bicolor* growth in AC conditions and only modest inhibition was observed in MC conditions (7.8 ± 2.8%). In fact, MS8a1 growth was inhibited by 15 ± 10% in AC and 39 ± 10% in MC. By the fifth day, however, both species were similarly affected. MS8a1's growth was reduced by 10 ± 7% in AC and 27 ± 9% in MC, while the growth of *L. bicolor* was reduced by 10 ± 4% in AC and 22 ± 10% in MC. By the day seven, MS8a1 significantly inhibited the growth of *L. bicolor* (36 ± 10% in AC and 32 ± 8% in MC), while the ECM had little effect on MS8a1 (5 ± 2% in AC and 15 ± 8% in MC). A similar behaviour was observed with the other *Trichoderma* strains (Supplementary Figs. S3C, S4C, S5C) upon cocultivation.

### Co-culture of *L. bicolor* and *Trichoderma* mutually lead to altered VOC emission profiles

The VOC analysis of *L. bicolor* and MS8a1 showed distinct profiles and emission strengths for the two species in solitary cultures, as shown by orthogonal partial least square regression discriminant analysis (OPLS-DA; CV-ANOVA =  < 0.001; 10.8% of VOC variance (Y axis) explained; Fig. 1B). For the other *Trichoderma* strains, WM24a1, ES8g1 and *T. atrobrunneum,* the differences in VOCs detected in PC compared to the co-cultures with *L. bicolor* explained 21.5%, 11.5% and 7.78% of the variance, respectively (Supplementary Figs. S3B, S4B, S5B).

#### Hierarchical clustering of VOC emissions by interaction type

Hierarchical clustering analysis (HCA) of VOCs from *L. bicolor* and MS8a1 in different degrees of contact revealed distinct clusters in PC compared to AC and MC (Fig. 1D). These clusters show individual, interaction-degree-specific VOC emission patterns (Supplementary Figs. S3D, S4D, S5D). Co-culture with different *Trichoderma* spp. resulted in the loss of putatively annotated VOCs originally detected in pure cultures of *L. bicolor*, such as p-cymene, nonanal and furfuryl alcohol and in a detection of new VOCs which were not detected in pure cultures. (Fig. 1D and Supplementary Figs. S3D, S4D, S5D, Supplementary Table S1).

#### Terpene emission profiles in pure cultures

**Fig. 2:**
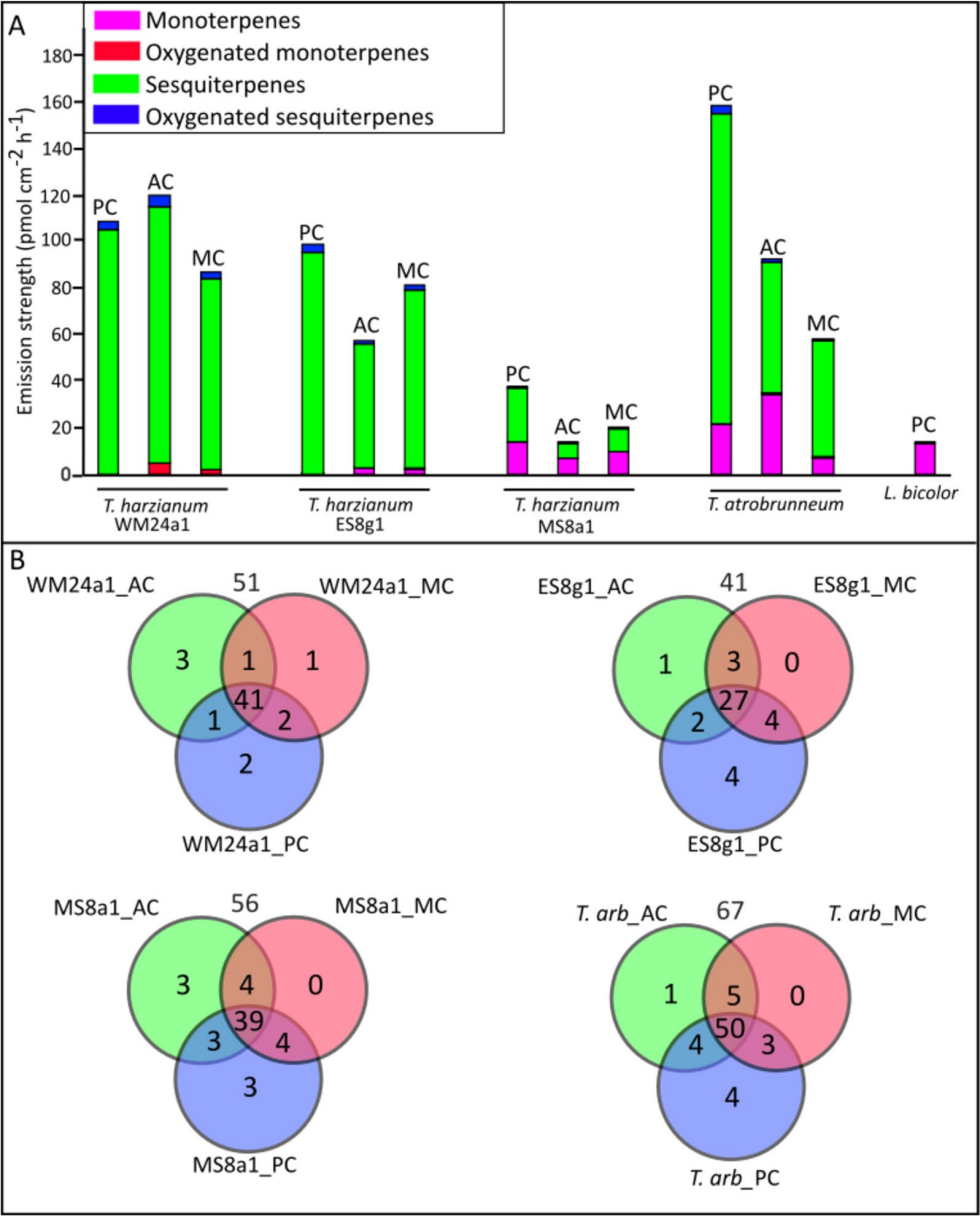
VOC profile of different fungal species. **A** The total emission of various Terpene VOCs across three *T. harzianum* strains (WM24a1, MS8a1 and ES8g1), *T. atrobrunneum (T. arb)* and *L. bicolor* grown as pure cultures (PC) and as co-cultures in aerial (AC) and media contact (MC). Values are an average of 5 replicates. **B** Venn plots depicting the total number of VOCs detected from the different *Trichoderma* strains across different growth and interaction conditions with *L. bicolor*. The number on top of each Venn plot indicates the total number of VOCs identified

Figure 2A (and Supplementary Table S1) shows the terpene VOC emission levels and patterns for each fungus in different culture conditions: in PC, 22 compounds for WM24a1, 21 for MS8a1, 26 for ES8g1, 32 for *T. atrobrunneum* and three for *L. bicolor.* MS8a1 showed the lowest terpene emission rate (38 pmol cm^2^ h⁻^1^), while *T. atrobrunneum* had the highest rate (181 pmol cm^2^ h⁻^1^). Although several unclassified and putatively annotated compounds were present in the VOC data (supplementary files), only the terpene VOCs were discussed in this section as they are the predominant class and can be putatively quantified based on the standards used.

#### Class distribution of VOCs during co-cultivation scenarios

*Trichoderma* spp. emission was mainly dominated by sesquiterpenes, while *L. bicolor* emitted mainly monoterpenes and very few sesquiterpenes. Co-culture significantly influenced VOC patterns and intensities compared to PC. There were only a few lost compounds (Fig. 2B), but the formation of new VOCs, i.e., those that were not found in pure cultures, was detected during co-cultivation scenarios (Fig. 2B), suggesting a possible role in intraspecific communication. Antagonistic confrontation (AC) between *L. bicolor* and *T. harzianum* (WM24a1) led to an overall increase in VOC emissions, whereas other *L. bicolor*—*Trichoderma* combinations resulted in reduced total emissions. Overall, both the appearance and loss of VOCs were highly species-specific (Fig. 2A). The core VOC profiles—defined as the set of compounds consistently detected across all co-cultivation scenarios—comprised 41 compounds for WM24a1, 27 for ES8g1, 39 for MS8a1, and 50 for *T. atrobrunneum*.

### Novel VOCs induced by co-culture

Co-cultivation of *L. bicolor* with various *Trichoderma* strains led to the emission of several novel VOCs, with strain-specific differences observed across interaction types. In the *L. bicolor*—WM24a1 interaction, co-culture during antagonistic confrontation (AC) induced four new VOCs: propylcyclopentane, 1-cyclopentylethanone, 2-methyl-5-propylthiophene, and 4-tert-butylphenol. Additionally, α-cuprene and 4-tert-butylphenol were newly detected during mycelial contact (MC). For the *L. bicolor—*ES8g1 pairing, although overall VOC emissions decreased, three novel compounds were identified during MC: 1-(dodecyloxy)−2-nitrobenzene, methylcycloheptane, and 1-methyl-4-trimethyl-cyclobenzene. The interaction between *L. bicolor and MS8a1* showed the lowest total VOC emissions, yet AC led to the emergence of three new compounds: heptamethyl-2-nonene, di-n-decyl ether, and 1,1,3,5-tetramethylcyclohexane. In co-culture with *T. atrobrunneum*, five novel VOCs were detected exclusively during AC, including 1-methyl-4-cyclohexene.

### Non-targeted metabolomics reveals strain- and species-specific metabolic diversity among *Trichoderma* and *Laccaria*

#### Establishing baseline metabolite profiles

**Fig. 3:**
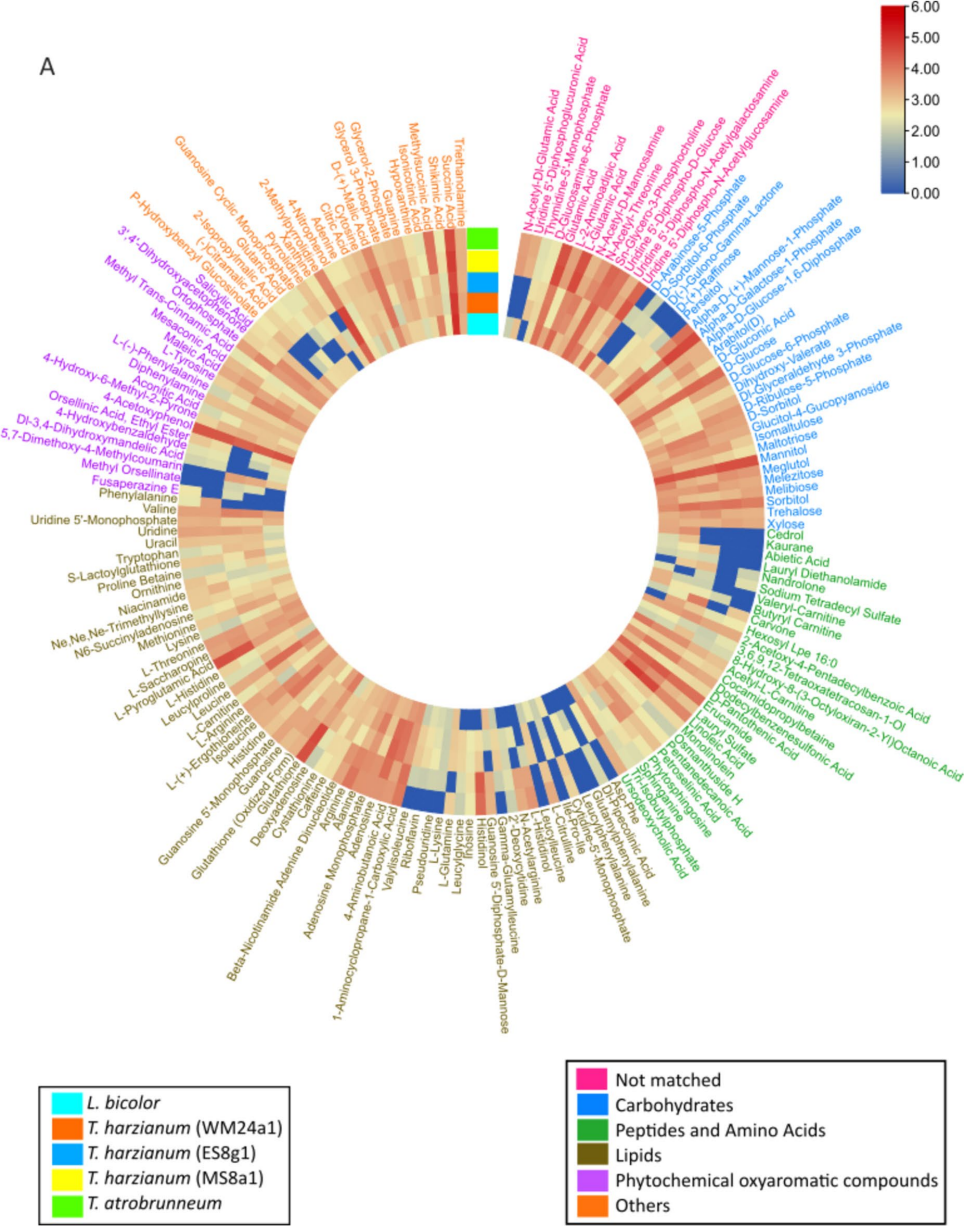

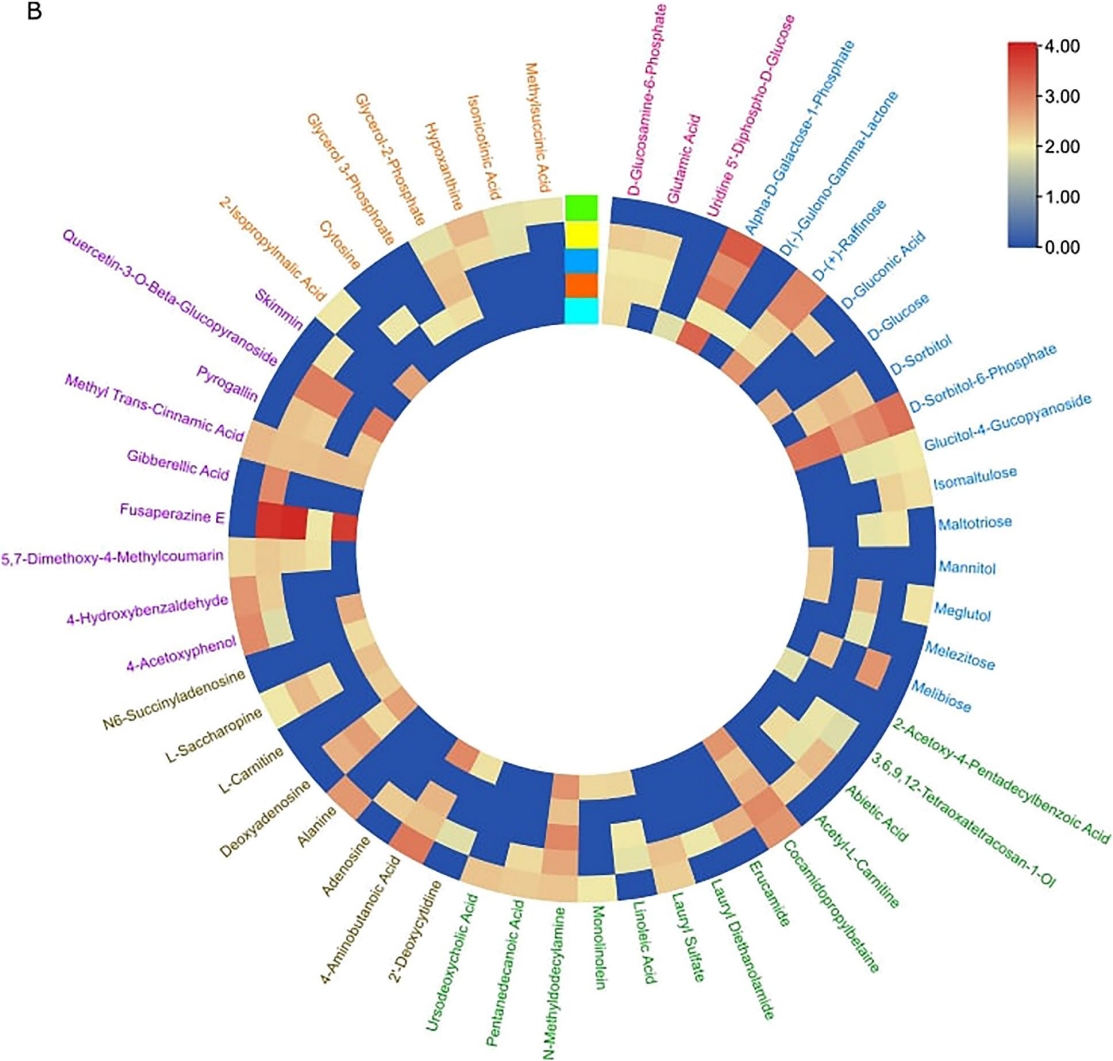
Circular heatmap of peak area of the metabolic data. The features detected in (**A**) fungal hyphae and (**B**) in the growth medium below the hyphae and cellophane. The fungi were grown as pure cultures. The metabolites found in pure media alone are subtracted in (**B**). The text colour indicates different chemical groups. Each block represents average values from 5 replicates

We analyzed total metabolite profiles of the hyphae and underlying media (exudates) of the pure cultures. These profiles served as baseline reference metabolomes that could be used for comparison with the hyphal metabolomes during co-culture, to detect metabolic shifts (Fig. 3A) and the secretion of potential signaling compounds into the medium (Fig. 3B).

We detected a total of 9365 mass features, of which 540 could be putatively identified (Level 2—matched to the spectral library), 7314 tentatively annotated features (Level 3—matched to the library using R codes and based on MS1 data [58]), 1450 metabolites matched to broad compound classes based on molecular formula predicted by MS1 data (Level 4 using the Multidimensional Stoichiometric Compound Classification (MSCC) approach) and 61 mass features of so far unknown unique features (Level 5) [58].

#### Chemical diversity among hyphae of the fungal species

The detected metabolites (Fig. 3A, shows only Level 2 annotated features) comprised a wide variety of different chemical classes, including amino sugars, carbohydrates, lipids, peptides and amino acids, phytochemicals and unclassified features. These metabolites were widely distributed among the different fungal species. While the carbohydrate composition of the fungal hyphae showed a striking similarity, strain-dependent variations were observed for lipids, peptides and phytochemicals (Fig. 3A). These findings highlight both conserved and divergent aspects of fungal primary and secondary metabolism. The total number of annotated and discriminatory mass features detected under each co-culture condition is provided in Supplementary Table S2.

#### Species-specific exudate profiles in culture medium

Analysis of the secreted metabolites in the medium, below the cellophane, revealed very diverse metabolite profiles for each spp. (Fig. 3B). Several features were secreted by all studied species, including the putatively annotated α-d-galactose-1-phosphate, d-raffinose, d-sorbitol, n-methyl-dodecylamine, cocamidopropyl betaine and methyltranscinnamic acid. Notably, certain features, including glutamic acid, abietic acid and *D*-sorbitol, were released exclusively by *T. harzianum* strains and were not detectable in *T. atrobrunneum* exudates. This indicates that the release of metabolites into the medium is both strain- and species-specific. The *L. bicolor* metabolome differed from that of *Trichoderma* strains, with features such as pyrolidine, thymidine, leucylglycin and histidinol not detected in *L. bicolor*, whereas they were found in all *Trichoderma* strains. Features such as cedrol, kaurene, *D*-sorbitol and butyl carnitine were unique only to *L. bicolor*.

#### Core metabolome categorization

Metabolic feature categorization revealed 1,921 and 1,687 metabolites as part of the core metabolome shared across all *Trichoderma* strains in hyphal tissues and culture medium, respectively (Supplementary Fig. S6). These conserved features provide a foundational metabolic baseline for interpreting species-specific differences and co-culture-induced variations.

### Co-culture of *Laccaria *and *Trichoderma* results in strain- and species-specific global alterations of hyphal metabolomes

To understand the chemical interactions between four *Trichoderma* strains/spp. and *L. bicolor,* we analyzed the metabolome patterns of hyphae in co-culture experiments, comparing to changes in PC (from Fig. 3). We considered two-time points: (i) early stage (day three) with no physical contact of hyphae but on a common media (media contact; MC) and (ii) later stage when direct contact (in DC) was established between hyphae (day five) (Fig. 1 and Supplementary Figs. S3, S4, and S5).

**Fig. 4:**
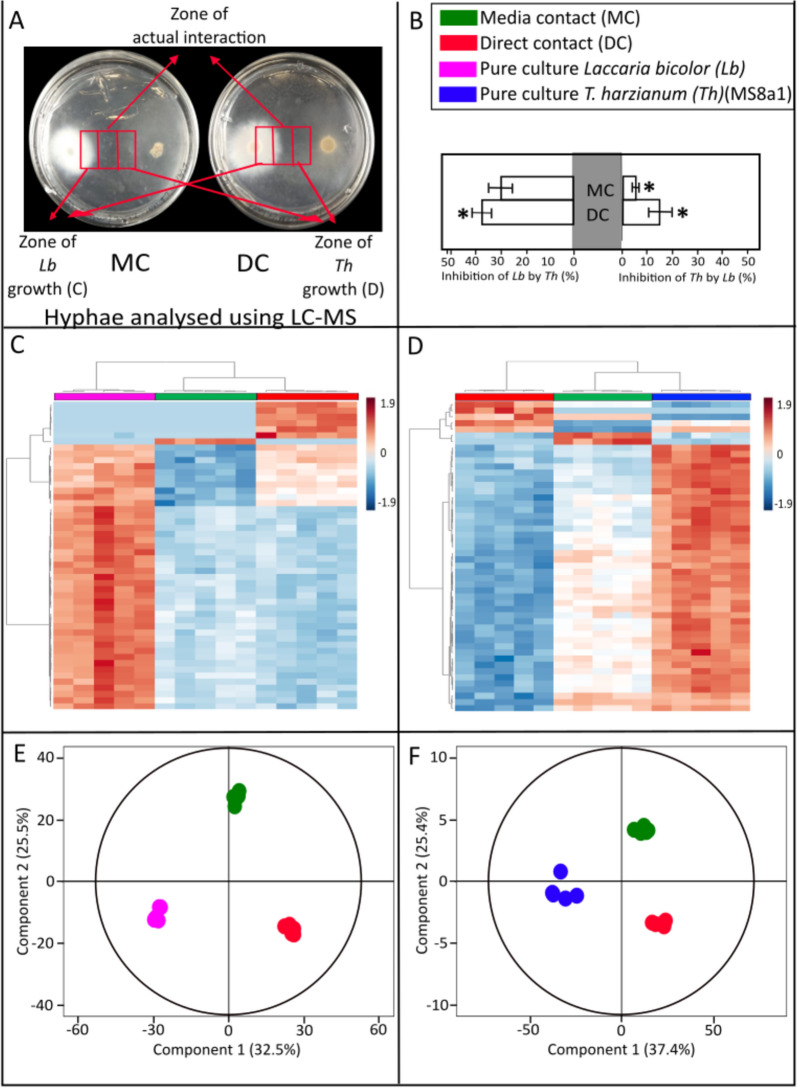
Metabolomic analysis of the hyphae from the co-cultivation experiment of *Lb* and *Th*. **A** Exemplary image of the confrontation assay showing the three different zones of sampling of hyphae and media across media contact (MC) and direct contact (DC). **B** Growth inhibition of *Lb* by *Th* (left) and vice versa (right) under different levels of co-cultivation compared to pure cultures. Significances are denoted as asterisks (one-way ANOVA and Tukey HSD, p < 0.05); mean ± SE; values are averages of 5 replicates. **C**, **D** Two-dimensional hierarchical clustering analysis of the peak area of top 50 discriminant features from cultures of (**C**) *Lb* and (**D**) *Th* hyphae. Features are selected based on having a Variable Importance of Projection (VIP) score > 1 to compute HCA. **E**, **F** Orthogonal partial least square regression discriminant analysis (OPLS-DA) showing differences among metabolic features under different levels of co-cultivation in (E) *Lb,* OPLS model fitness: R^2^X(cum) = 0.953, R^2^Y(cum) = 1, Q^2^Y(cum) = 0.961, CV-ANOVA = 1.89 × 10^–8^ and (F) *Th* OPLS model fitness: R^2^X(cum) = 0.977, R^2^Y(cum) = 1, Q^2^Y(cum) = 0.95, CV-ANOVA = 4.97 × 10^–11^

Growth inhibition results reflect interaction intensity of the two fungal strains in contact, MS8a1 and *L. bicolor* (Fig. 4). In general, co-culture showed a resembling pattern of growth inhibition as shown in Fig. 1. The strain MS8a1 showed a stronger inhibitory effect on *L. bicolor* in DC (40 ± 8%) than MC (30 ± 5%), whereas *L. bicolor* weakly inhibited the growth of MS8a1, reducing it by 7 ± 3% in MC and 16 ± 5% in DC (Fig. 4B). Other *Trichoderma* strains (WM24a1, ES8g1, *T. atrobrunneum*) exhibited a similar behavior, with stronger growth inhibition of *L. bicolor* (Supplementary Figs. S7B, S8B, and S9B).

#### Global metabolic changes in co-culture

Metabolomic profiling by HCA and OPLS-DA demonstrated clear distinctions between PC and co-cultured hyphae. Hierarchical cluster analysis and OPLS-DA for MS8a1 (Fig. 4D and F) and *L. bicolor* (Fig. 4C and E) showed distinct metabolic patterns between PC and co-cultured mycelia. The heatmaps demonstrated global changes in the hyphal metabolomes due to interactions between MS8a1 and *L. bicolor*. The metabolic profiles allowed differentiation of scenarios along predictive component 1 (PC1; 32.5% for *L. bicolor* and 37.4% for MS8a1) and between MC and DC along PC2 (25.5% for *L. bicolor* and 25.4% for MS8a1). Similarly, confrontation scenarios between *L. bicolor* and the other *Trichoderma* strains—WM24a1 (Supplementary Fig. S7), ES8g1 (Fig. S8), and *T. atrobrunneum* (Fig. S9)—were statistically distinguishable based on their metabolic profiles. This suggests that each strain and species developed distinct, interaction-dependent metabolic fingerprints.

Statistical analysis revealed distinct metabolic responses in both interacting fungi, highlighting several features potentially associated with defence mechanisms or growth regulation. In the MC interaction between *L. bicolor* and MS8a1, 493 discriminant mass features were identified, of which five showed an increased concentration and 488 decreased in concentration compared to pure cultures. *L. bicolor* showed 423 discriminant features, of which 22 were increased and 401 decreased in concentration (Supplementary Table S2). In direct contact, MS8a1 hyphae had 478 discriminatory features, with numbers of 319 decreased and 159 increased mass features. On the other hand, *L. bicolor* had 350 discriminatory features, of which the concentration of 254 was decreased and of 196 was increased, respectively.

#### Putative annotations of common significant metabolites

Among the mass features that showed reduced concentration during co-cultivation, only a limited number of shared features could be putatively annotated at Level 2 confidence. These include amino butanoic acid, malic acid, valine and triethanolamine, which appear to be present in all *T. harzianum* strains under MC. In DC features such as L-tyrosine, uridine, lauryl sulphate, perseitol, glutathione, D-ribulose-5-phosphate and L-arabitol were common to all *T. harzianum* strains.

### Co-culture leads to species-specific enrichment of metabolic pathways with different functions in growth and defence

**Fig. 5:**
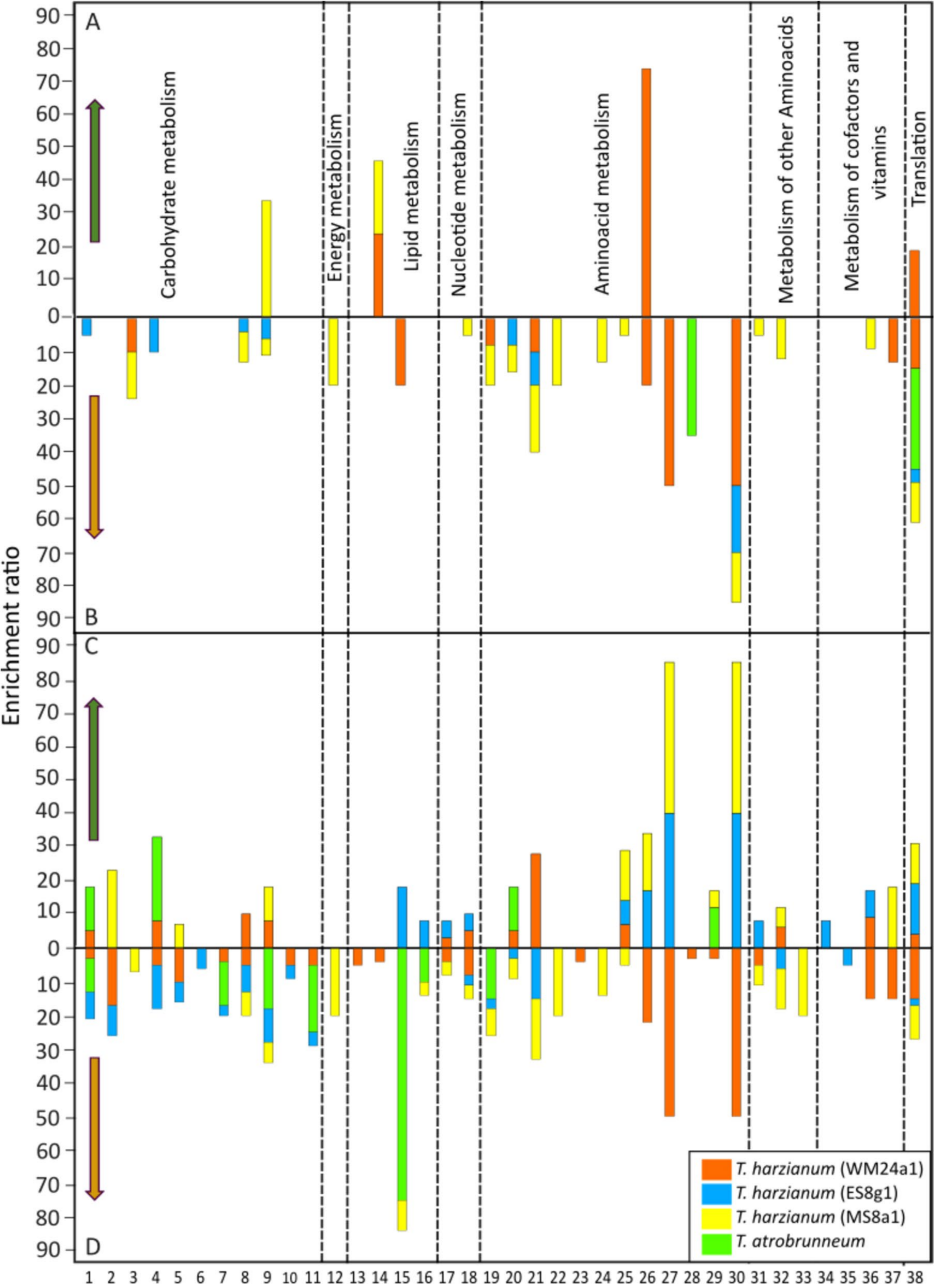
The enrichment ratios of pathways identified by significant metabolic features. The ratios are based on putative annotation in hyphae of different *Trichoderma* spp. in co-cultivation with *L. bicolor* (*Lb)* and significantly (**A**) increased and (**B**) decreased in concentration in media contact (MC) and (**C**) increased and (**D**) decreased in direct contact (DC). The numbers 1 to 38 indicate different pathways as listed in the Table 1. Pathways are grouped according to metabolic processes

To gain a comprehensive understanding of the pathway regulation during the interaction between *Trichoderma* spp. and *L. bicolor,* we calculated the enrichment ratios of putatively annotated metabolites in the different pathways (Fig. 5). A total of 38 metabolic pathways were statistically significant in the hypergeometric test (p ≤ 0.05) and identified as being either increased or decreased abundance in the hyphal cells during the antagonistic interaction between *L. bicolor* and *Trichoderma* spp*.* The changes observed in both *L. bicolor* and *Trichoderma* spp. were highly species- and strain-specific. Overall, the antagonistic interactions resulted in a generalized increased abundance, which was only offset by an increased abundance of metabolites related to only a few pathways. The affected pathways are fundamental for cellular processes and growth being involved in metabolism of carbohydrates, energy, lipids, nucleotides, amino acids, cofactors, and vitamins, as well as in translation.

**Table 1:** Pathways enriched by putatively annotated and significant metabolic features grouped according to metabolic processes

ID	Pathway	Process
1	Amino sugar and nucleotide sugar metabolism	Carbohydrate metabolism
2	Ascorbate and aldrate metabolism	
3	Butanoate metabolism	
4	Fructose and mannose metabolism	
5	Galactose metabolism	
6	Glycolysis	
7	Glyoxalate and dicarboxylate metabolism	
8	Pentose and glucuronate interconversions	
9	Pentose phosphate pathway	
10	Starch and sucrose interconversions	
11	TCA cycle	
12	Nitrogen metabolism	Energy metabolism
13	Ether lipid metabolism	Lipid metabolism
14	Glycerophospholipid metabolism	
15	Linolenic acid metabolism	
16	Unsaturated fatty acid biosynthesis	
17	Purine metabolism	Nucleotide metabolism
18	Pyrimidine metabolism	
19	Alanine, asparate and glutamate metabolism	Amino acid metabolism
20	Arginine and proline metabolism	
21	Arginine biosynthesis	
22	Glutamine and glutamate metabolism	
23	Glycine, serine and threonine metabolism	
24	Histidine metabolism	
25	Lysine degradation	
26	Phenylalanine metabolism	
27	Phenylalanine, tyrosine and tryptophan biosynthesis	
28	Tryptophan metabolism	
29	Tyrosine metabolism	
30	Valine, leucine and isoleucine biosynthesis	
31	Beta-alanine metabolism	Metabolism of other amino acids
32	Glutathione metabolism	
33	Phosphonate metabolism	
34	Biotin metabolism	Metabolism of cofactors and vitamins
35	Nicotine and nicotinamide metabolism	
36	Pantothenate and CoA biosynthesis	
37	Ubiquinone biosynthesis	
38	Aminoacyl T-RNA biosynthesis	Translation

#### Pathway regulation under media contact (MC)

In MS8a1, metabolites related to glycerophospholipid and pentose pathways were increased in concentration in response to *L. bicolor*, with enrichment ratios of 22 and 35, respectively. *T. harzianum* (MS8a1) exhibited reduced abundance in multiple metabolic pathways, including three related to carbohydrates, seven to amino acids, and one each in nitrogen metabolism, pyrimidine metabolism, β-alanine metabolism, glutathione metabolism, and the pantothenate and CoA biosynthesis pathway, with varying levels of enrichment (Fig. 5B).

In the context of mycelial contact (MC), only the *Trichoderma* strains WM24a1 and MS8a1 displayed increase in regulation of metabolic pathways. The greatest extent of increased abundance was observed in the metabolites related to phenylalanine pathway in WM24a1, with an enrichment ratio of 75. Metabolites related to glycerophospholipid metabolism and aminoacyl-tRNA biosynthesis were also increased in abundance, with enrichment ratios of 25 and 20, respectively (Fig. 5A).

Conversely, putatively annotated features that decreased in concentration were found to be enriched in the aminoacyl-tRNA biosynthetic pathway in MC. *T. atrobrunneum* showed an additional decreased metabolite abundance in the tryptophan metabolism. All three *T. harzianum* strains (WM24a1, MS8a1 and ES8g1) showed a reduced abundance of features enriched in arginine as well as valine, leucine and isoleucine biosynthesis.

*T. harzianum* (WM24a1) showed decreased abundance across several metabolic pathways, including butanoate metabolism, linolenic acid metabolism, alanine, aspartate and glutamate metabolism, phenylalanine, tyrosine and tryptophan biosynthesis, as well as ubiquinone biosynthesis. Similarly, *T. atrobrunneum* exhibited a reduced abundance in four carbohydrate metabolism pathways and three amino acid metabolism pathways (Fig. 5B).

#### Pathway regulation under direct contact (DC)

In the DC scenario, significantly fewer metabolites related to metabolic pathways were detected to be increasing in abundance than decreasing. For MS8a1, we observed an increased abundance of metabolites involved in three pathways related to carbohydrate metabolism and six each in amino acid- and ubiquinone metabolism (Fig. 5C). WM24a1 showed an increased abundance of metabolites in four pathways involved in carbohydrate metabolism, five in amino acid, glutathione, pantothenate and CoA metabolism, whereas ES8g1 exhibited an increased abundance of metabolites in six pathways including those involved in amino acid, pantothenate and CoA metabolism, as well as two pathways related to lipid and nucleotide metabolism. In *T. atrobrunneum*, only a subset of pathways was enriched by the mass features increasing in concentration, including fructose, mannose, arginine, proline and tyrosine metabolism (Fig. 5C).

#### Global pathway suppression patterns upon direct contact

Upon DC, a significant increase in the total number of putatively annotated mass features and enriched pathways was observed for all examined species. All *Trichoderma* spp. showed differential expression of features enriched in aminoacyl-tRNA biosynthesis. MS8a1 exhibited reduced abundance of metabolites linked to three pathways related to carbohydrate metabolism, nine related to amino acid metabolism, one related to nitrogen metabolism, two related to lipid metabolism and two related to nucleotide metabolism.

WM24a1 showed a reduced abundance of metabolites in eight metabolic pathways related to carbohydrate metabolism, seven pathways related to amino acid, glutathione and pantothenate metabolism and two pathways related to lipid and nucleotide metabolism. Also in the other *Trichoderma* strains reduction of metabolite abundance were linked to reduction in pathways: These were for ES8g1 10 pathways related to carbohydrate metabolism, three related to amino acid metabolism, one related to nucleotide metabolism and one related to pantothenate metabolism and for *T. atrobrunneum* four related to carbohydrate metabolism, two to lipid metabolism and one pathway to amino acid metabolism (Fig. 5D).

### Laccaria and *Trichoderma* spp. hyphal exudates change during antagonism

**Fig. 6:**
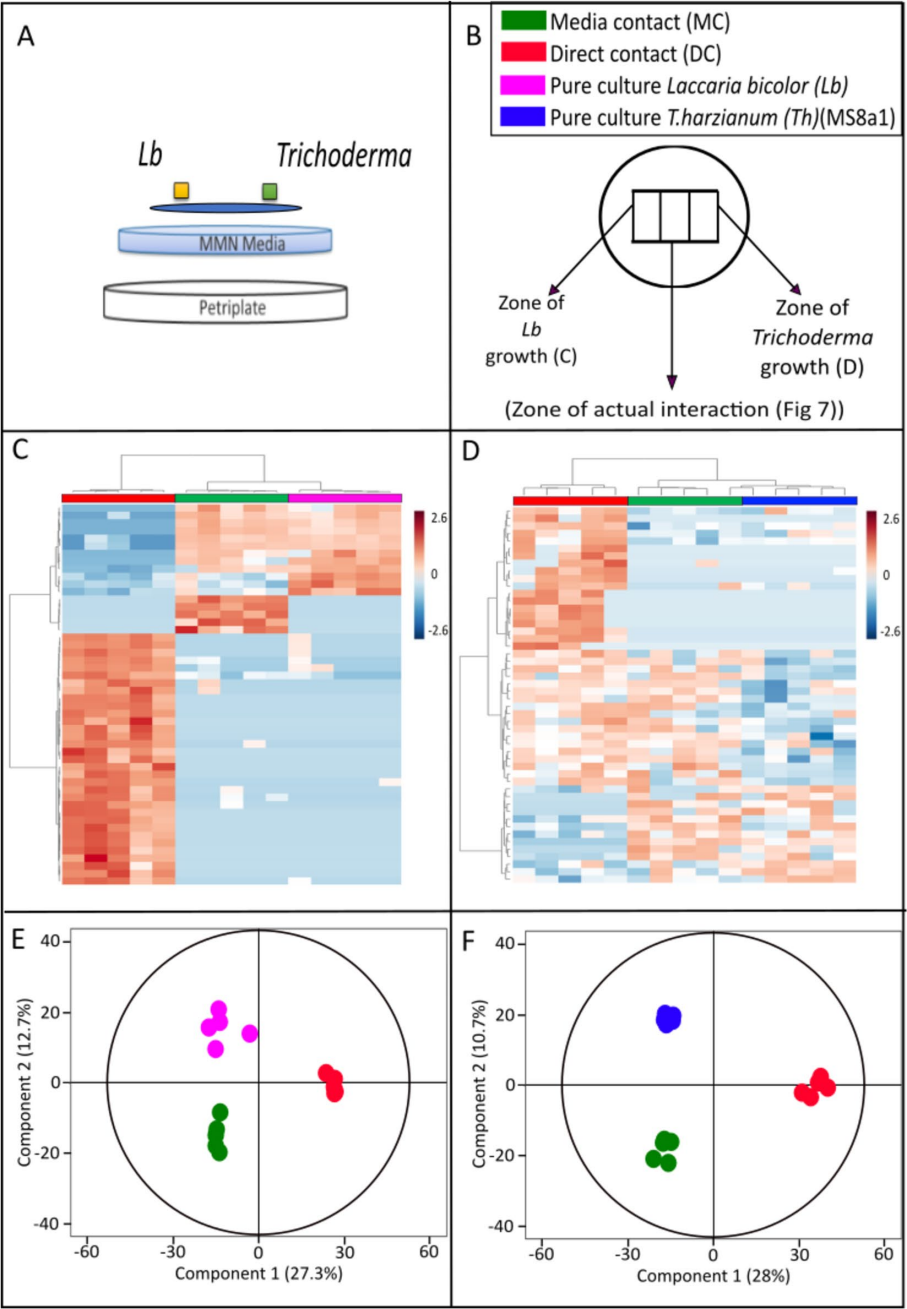
Metabolomic analysis of the media from the co-cultivation experiment of *Lb* and *Th.* **A** Schematic representation of the co-cultivation experiment between *Lb* and *Th* showing the position of fungal inoculants from the two species, cellophane sheet and the underlying media. **B** Experimental set-up of the confrontation assay showing the three different zones of sampling of media across media contact (MC) and direct contact (DC). **C**, **D** Two-dimensional hierarchical clustering analysis of the peak area of top 50 discriminant features from cultures of (**C**) *Lb* and (**D**) *Th.* **E**, **F** Orthogonal partial least square regression discriminant analysis (OPLS-DA) showing differences among metabolic features in media under different levels of co-cultivation in (E) *Lb,* OPLS model fitness: R^2^X(cum) = 0.953, R^2^Y(cum) = 1, Q^2^Y(cum) = 0.799, CV-ANOVA = 6.45 × 10^–4^ and (F)*Th,* OPLS model fitness: R^2^X(cum) = 0.98.7, R^2^Y(cum) = 1, Q^2^Y(cum) = 0.785, CV-ANOVA = 4.23 × 10^–4^

The mycelia are in contact with the culture medium through cellophane, via which they absorb nutrients, minerals and water. The hyphae in turn secrete soluble metabolites into the medium (Fig. 6). Thus, in addition to the gas phase (Figs. 1, 2), the medium is one of the ways in which the two fungi could communicate. To gain further insight into the complex chemistry involved in the interaction between the two plant beneficial fungi, we developed an experimental setup to analyze the metabolic composition of the chemical exudates (Fig. 6A).

We analyzed culture media segments i) directly beneath the hyphae ii) in the interphase between the fungi before direct physical contact (MC) and later iii) directly below the hyphae of the two fungi in DC with each other (zone of interaction). After removal of the cellophane between the hyphae and media, the metabolic exudates from the three zones were analyzed (Fig. 6B).

#### Interaction-specific metabolic patterns

The HCA and OPLS-DA analyses revealed different metabolite patterns depending on the degree of interaction between *Laccaria* (Fig. 6C, E) and MS8a1 (Fig. 6D, F). Similarly, clear pattern differences in the metabolic profiles were observed for the other *T. harzianum* strains (Supplementary Figs. S10, S11) and for *T. atrobrunneum* (Supplementary Fig. S12). *L. bicolor* also responded individually to the presence of each of the *Trichoderma* species, as evidenced by changes in the metabolite secretion. The observed changes in the metabolite pattern of *L. bicolor* were dependent on the partner strain/species encountered, suggesting a specific, strain-dependent exudation response in the ECM fungus (Fig. 6, and Supplementary Figs. S10, S11, S12).

All the heatmaps based on the *Laccaria*-*Trichoderma spp*.-interaction metabolites showed distinct patterns in MC (Fig. 6C and D, and Supplementary Figs. S10, S11, S12). The OPLS-DA demonstrated a clear separation of the metabolic profiles between the exudates of the pure cultures and the co-culture scenarios along PC2 (12.7% for *L. bicolor* and 10.7% for MS8a1 and between MC and DC scenarios along PC2 (27.3% for *L. bicolor* and 28% for MS8a1 (Fig. 6E and F). Clear separation in the OPLS-DA was also evident in the confrontations of *L. bicolor* with *T. harzianum* (WM24a1 and ES8g1) and with *T. atrobrunneum* (Supplementary Figs. S10, S11, S12, C and D).

#### Discriminating metabolites in the interaction zones

Of the 92 discriminating mass features in the medium under the mycelium of MS8a1, 22 showed increased and 70 showed decreased concentrations. In *L. bicolor* samples in direct contact with MS8a1 only 78 discriminating metabolites were detected in the exudate with the concentration of 19 features increasing and 59 decreasing.

Direct contact resulted in 159 discriminating mass features under the MS8a1 mycelium, with 47 features showing increasing concentrations and 112 features showing decreasing concentrations. Under MC in *L. bicolor* mycelium, only 15 features with decreasing concentrations were detected, which may be due to the overgrowth by MS8a1 hyphae.

#### Comparison across strains and interaction stages

The total number of discriminatory features under each co-culture condition for each fungus examined is shown in Supplementary Table S3. While not many differentially expressed features could be annotated, the highest increase in abundance of metabolic features was observed under MS8a1 mycelium in DC with *L. bicolor*. Compared to the other *Trichoderma* strains*, T. atrobrunneum* exuded fewer active metabolites with only four and three features increasing in concentration in DC and MC, respectively. Fewer differentially regulated features were also detected from *L. bicolor* in DC than in other co-culture scenarios, suggesting that *L. bicolor* released generally fewer features towards the end of the co-culture with *Trichoderma* spp. (Supplementary Table S3).

### Exudate composition depends on the distance between the interacting fungi

**Fig. 7:**
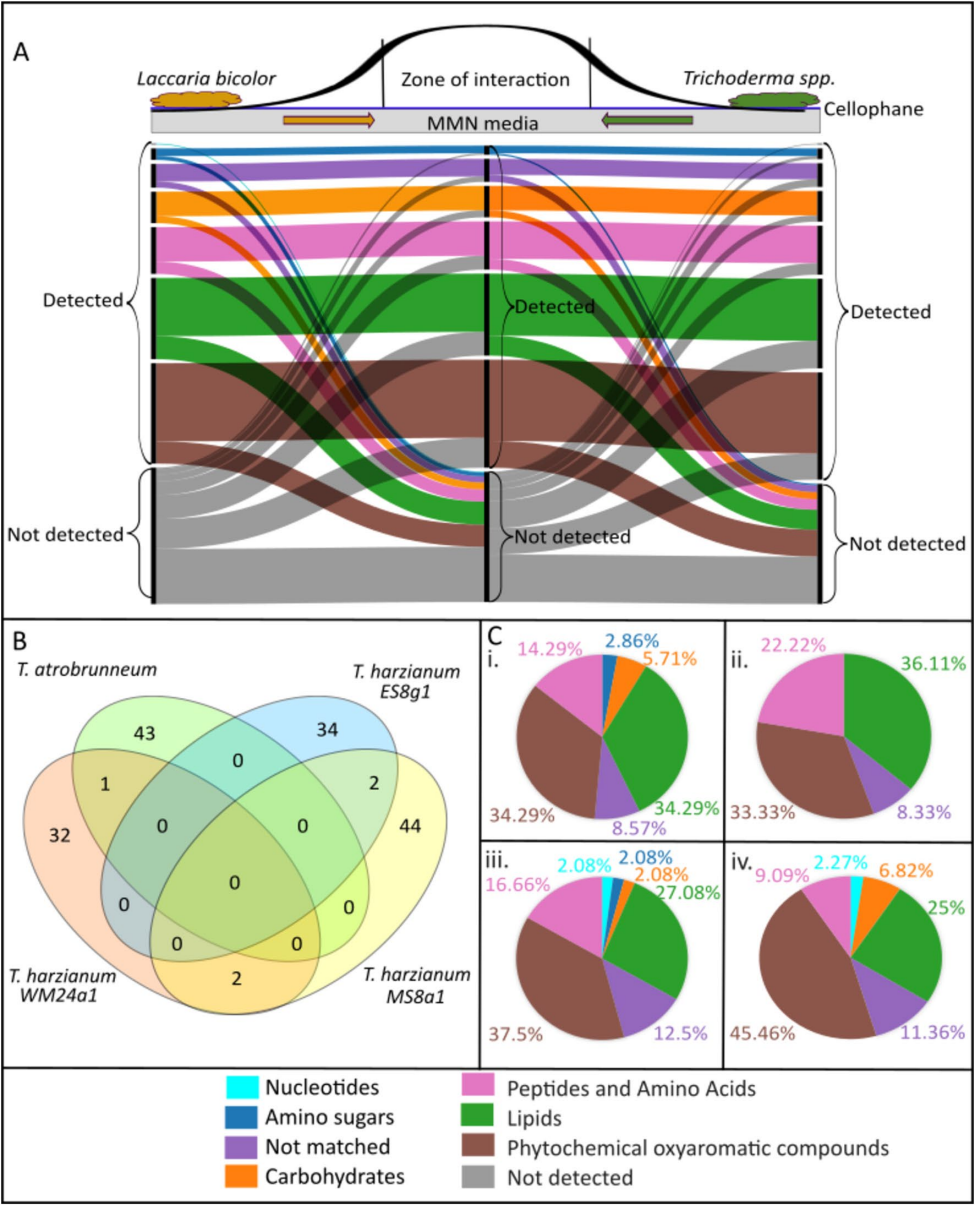
Metabolomic analysis of the media from the zone of actual interaction between *Lb* and *Th.* **A** Representative alluvial plot showing the flow of exudates from *L. bicolor* to *T. harzianum (*MS8a1*)* and vice versa through the common growth media in co-cultivation (read from left to right). The colour patterns depict features belonging to different chemical groups classified by ‘multidimensional stoichiometric compound classification’ (MSCC) if detected in a particular zone. **B** Venn plot depicting the exudates found ‘uniquely’ in the zone of interaction across four different species. **C** Chemical class classification of the features by MSCC of the features found ‘only’ in the zone of interaction across co-cultivation of (i) *T. harzianum* (WM24a1), (ii) *T. atrobrunneum,* (iii) *T. harzianum* (ES8g1) and (iv) *T. harzianum* (MS8a1) with* L. bicolor*

The composition of diffusible metabolites included nucleotides, amino sugars, carbohydrates, peptides and amino acids, lipids and phytochemical oxyaromatic compounds for all the studied fungi analyzed at MC (Fig. 7). An alluvial plot (color patterns from left to right) allowed analysis of the flow and diffusion of exudates from each fungus into the contact zone. The analysis revealed six distinct, species-dependent patterns of mass flow for all *Laccaria*—*Trichoderma* interactions (Fig. 7A). The features were classified into chemical groups only when detected (otherwise reported as not detected).

Overall, the distinguishing mass features in MS8a1 exudates were predominantly oxyaromatic compounds, followed by lipids, peptides and carbohydrates. In all other *T. harzianum* strains (WM24a1, ES8g1 and *T. atrobrunneum*), the predominant secreted compounds were lipids, followed by oxyaromatic compounds, peptides and carbohydrates (Fig. 7C).

#### Spatial variability in mass feature distribution

The relative abundance of individual mass features in the interaction zone often differed from those observed directly beneath the mycelium each fungus. In several instances, features detected under the fungal mycelium were absent in the interaction zone, or entirely new features emerged exclusively within the interaction zone alone. For example, in the case of *L. bicolor* and MS8a1, many of the mass features that were detected directly below the fungus were not found in the interaction zone (Fig. 7A). The analysis of the exuded metabolites of *T. harzianum* (WM24a1, ES8g1) and *T. atrobrunneum* in interaction with *L. bicolor* showed similar results: the composition of the detected mass features was different directly below the fungus compared to the interaction zone (Supplementary Fig. S13). In addition to the species-specific exudation patterns in *Trichoderma* spp, we also observed interaction partner-dependent differences in the exudation of *L. bicolor* (Fig. 7A and Supplementary Fig. S13).

#### Accumulation of newly diffused metabolites

Differences in metabolite patterns became especially pronounced when analysing the accumulation of newly diffused metabolites detected exclusively in the interaction zones, compared to the corresponding same spatial regions in pure culture controls (PC). The composition of accumulated metabolites in the interaction zone differed between *L. bicolor* and the four *Trichoderma* confrontation experiments. All three *T. harzianum* strains (WM24a1, MS8a1 and ES8g1) and *T. atrobrunneum* showed unique metabolic mass characteristics in the interaction zone, with 32, 44, 34 and 43 features, respectively (Fig. 7B). WM24a1 and MS8a1, as well as MS8a1 and ES8g1, each shared two common mass features. In contrast, only one common mass feature was detected between WM24a1 and *T. atrobrunneum* (Fig. 7B). However, these common features could not be annotated.

#### Antifungal and strain-specific metabolites in the zone of interaction

Some of the mass features detected in the interaction zone could be putatively annotated as phenolic compounds with antifungal properties [68–70]. These features included quercetin 3-O-rutinoside in *T. harzianum* (WM24a1) interaction zone, salicylic acid and pyrogallin in *T. harzianum* (ES8g1) interaction zone and vanillin in *T. harzianum* (MS8a1) interaction zone. We detected moreover some metabolites that were specific for *T. harzianum* strains and were not detected in *T. atrobrunneum* interactions. Such features, detected exclusively in the interaction zone of *L. bicolor*—*T. harzianum* strains, included minimal amounts of nucleotides, amino sugars and carbohydrates.

## Discussion

### Restricted mycelial growth and altered VOC profiles in *L. bicolor *– *Trichoderma* co-cultures

Due to their volatility and diffusivity, VOCs are the ideal signalling molecules between interacting fungi even before direct hyphal contact [17]. To function as signalling molecules, unique, adaptable VOC profiles are considered as a prerequisite [71]. Our study reveals highly unique VOC profiles under solitary cultures but also under dual cultivation for all species and interaction scenarios investigated. The profiles were, furthermore, adapted depending on the degree of contact with other fungal species, thus suggesting function in fungal interaction and supporting our first hypothesis (I). Unique VOC profiles were previously detected also from different *T. harzianum* strains in pure cultures [27, 72–75]. For example, Lee et al. [72] detected 27 VOCs from *T. harzianum* CBS 227.95 and Siddiquee et al. [75] even 278 VOCs from the *T. harzianum* strain FA1132. Moreover, when three other *Trichoderma* species (*T. hamatum* QL15d1, *T. reesei* QM6a and *T. velutinum* GL1561) were studied as pure cultures and in co-culture with *L. bicolor,* highly unique, species and interaction specific VOC profiles were reported [10].

In accordance with previous results [10], the present results show that the interaction of *Trichoderma* spp. with *L. bicolor* leads to strong interaction-dependent adjustments in the VOC profiles even if headspace VOC concentrations tended to be reduced in dual cultures compared to PC. An exception was the WM24a1—*L. bicolor* interaction in AC with increased concentration of overall VOC emission, which also appears to be special in other respects: Transcriptomic data of Stange and colleagues [16] showed that in a similar co-culture set-up, where communication was only allowed through VOCs, biochemical (KEGG) pathways associated with the biosynthesis of secondary metabolites were increasing in abundance in *L. bicolor.* The authors observed a downregulation of WM24a1 terpene synthase gene (M431DRAFT_113113). In line with these results, in the present study, *L. bicolor* growth was overall only marginally affected in AC with *Trichoderma* and especially WM24a1, suggesting that the *Trichoderma* VOCs have no or only a weak effect on *L. bicolor* growth, whereas ECM VOCs may negatively affect *Trichoderma* performance. At later stages, however, when also exchange of soluble metabolites were allowed, *Trichoderma* behaved more aggressively by overgrowing *L. bicolor* under in vitro conditions. Somewhat contradictory to the growth measurements, that revealed inhibition of *Trichoderma* growth in the presence of *Laccaria* VOCs, in general reduced *L. bicolor* VOC profiles were observed in co-cultures. The results suggest that either the ECM’s VOC compounds are somehow degraded in the presence of *Trichoderma* spp. or, alternatively, *L. bicolor* may simply release other VOCs or invests more in other, soluble metabolites instead of VOCs when sensing a competitor. Growth inhibition may also be a result of fungal confrontation during the sensing phase rather than from the influence of another interacting species. However, Stange et al. [16] conducted *Trichoderma* self-controls with the same strains as used here but did not observe any inhibition. The growth inhibition trends of a confrontation in that work were found to be similar as in the present study. Together the present results of fungus-specific and interaction degree-dependent changes in VOC profiles strongly support the hypothesis that the studied fungi can regulate their VOC emissions in response to environmental constraints. This adjustability suggests that fungal VOCs possess important ecological functions in microbial interactions and perception.

### Altered metabolic responses in the hyphae and exudates of *Trichoderma* and *L. bicolor* in co-cultures

Analyses of core metabolomes are essential to understand the fungal responses to various environmental changes. The present metabolite analyses highlight pronounced similarities among *T. harzianum* strains and species-specific metabolic diversity with *T. atrobrunneum*. A comprehensive metabolite analysis of hyphae and exudates in pure cultures identified 9365 mass features with varying levels of putative annotation (Level 2–4). Given the exploratory nature of our study, we relied on putative annotations (Levels 2–4), which were based on MS/MS libraries and elemental composition. While these provide meaningful insights into changes at the level of chemical classes, further validation using authentic standards is necessary to confirm the identities of individual compounds. While this limits the exploration of individual metabolites, it allows the classification of functional chemical composition. Our hypothesis (I), that there is a high degree of metabolic similarity within the studied *Trichoderma* spp., is however, only weakly supported by the data: While similar carbohydrate composition was detected in the hyphae of the different *T. harzianum* strains, there were notable strain-dependent differences in lipids, peptides and phytochemicals. Secreted metabolite profiles also varied: features such as α-*D*-galactose-1-phosphate and *D*-raffinose were common to all studied fungi whereas, in the hyphae of the studied ECM unique metabolites such as cedrol, kaurene, *D*-sorbitol and butyl carnitine were detected. The core metabolomes can serve as reference metabolome to identify the changes in hyphal and exudate composition that may facilitate chemical exchange between interacting fungi on co-cultivation.

*Trichoderma* species are known to produce exudates that degrade microbial cells in soil habitats, altering the ability of other species to absorb nutrients and persist [76]. *Trichoderma harzianum,* for example, can alter plant pathogenic fungal communities and some of its metabolites (incl. harzianopyridone, pyrone and trichorzianine) show antifungal activity [77–79]. How *Trichoderma* spp. interacts with non-plant pathogenic fungi is less well understood. Our study reveals for the first-time insights into the soluble metabolites potentially involved in interactions between *Trichoderma* spp. and an ECM*.* The results show that co-culture with the *L. bicolor* leads to distinct strain- and species-specific changes in the exudates and in the hyphal metabolites of *Trichoderma*. These alterations include global changes that distinguish pure cultures from co-culture scenarios (MC and DC) in all studied *Trichoderma* strains, suggesting interaction-dependent metabolic fingerprints. The altered metabolome may have also directly influenced the fungal growth: *Trichoderma* constantly inhibited *L. bicolor* growth more than vice versa when the exchange of soluble metabolites was allowed.

Some common features were detected in the hyphal metabolome of the different *T. harzianum* strains, including the increased abundance of such features as L-tyrosine, uridine, lauryl sulfate, perseitol, glutathione, *D*-ribulose-5-phosphate and L-arabitol in DC. In MC all the studied *T. harzianum* strains showed reduced abundance of aminobutanoic acid, malic acid, valine and triethanolamine. These results highlight that the metabolic responses that occur upon interaction with other fungi are at least to some extent shared among the different strains. However, whereas the metabolites detected within the hyphae are interesting allowing direct insight into the altered fungal metabolism, the metabolites secreted into the growth matrix might play a more critical role in fungal interactions. Statistical analysis revealed many differentially regulated metabolic features in the *Trichoderma* exudates. Many of these were previously assigned a role in fungal defence or inhibition (discussed below). The differential regulation suggests specific chemical interactions and activated defence mechanisms upon sensing a competitor. Striking were also the notable differences detected in the exudate profiles of *L. bicolor* confronted with different *Trichoderma* spp. These findings suggest that the ECM can sense the competitor and show a unique exudation response towards a specific *Trichoderma* spp. However, previous studies on interaction between soil fungal communities also suggest that *Trichoderma* species can distinguish between fungal partners with different lifestyles—beneficial ectomycorrhizal fungi (represented by *Laccaria bicolor* and *Hebeloma cylindrosporum*) and pathogenic fungi (*Fusarium graminearum* and *Alternaria alternata*)—in different confrontation scenarios at a distance [16]. The results challenge the hypothesis that there would be a conserved exudation response in *L. bicolor* towards different antagonistic fungi. Although fixed sampling time points were used to ensure comparability across treatments, we acknowledge that varying degrees of growth inhibition may have influenced the developmental stage of each fungus, potentially confounding some metabolic readouts. Future studies employing biomass-normalized sampling or time-resolved profiling could help disentangle growth-related effects from interaction-specific metabolic shifts.

### Co-cultivation led to altered metabolic pathway enrichment influencing growth and defence during fungal interactions

As the fungi grow towards each other, they continuously sense their environment and initiate specific downstream pathways based on the prevailing abiotic and biotic conditions [80–84]. Enrichment analysis of the putatively annotated metabolic features (level 2) revealed differential regulation of several metabolic pathways involved in carbohydrate, energy, lipid, nucleotide, and amino acid metabolism. Altogether, we identified 38 metabolic pathways that were altered during the antagonistic interactions, although decreasing abundance was observed more frequently than increasing abundance. For example, all *T. harzianum* strains exhibited a reduction in pathways associated with arginine and valine, leucine and isoleucine biosynthesis on MC. These alterations together with observed reduced growth may reflect the increased investment to defence or interaction related metabolites and less to growth. In general, such response of *T. harzianum* strains was stronger compared to *T. atrobrunneum*, former showing more metabolic pathways enriched by features decreasing in concentration.

Metabolic pathways with features increasing in abundance in the DC scenario were significantly fewer than decreasing ones. A few commonalities among the pathways with increasing abundance were found especially among the *T. harzianum* strains, though. For example, MS8a1, WM24a1 and ES8g1 all showed increased abundance in pathways related to amino acid metabolism, whereas in MS8a1 and WM24a1 carbohydrate metabolism related pathways were altered. Ubiquinone metabolism related changes were, however, specific to MS8a1 and pantothenate and CoA metabolism related increase in abundance was specific to ES8g1. In *T. atrobrunneum,* only a subset of pathways was enriched by the mass features increasing in concentration eventually reflecting less aggressive defence reaction compared to *T. harzianum* strains in the presence of *L. bicolor*.

Together the detected alteration in various pathways shows that the interacting fungi can sense each other through signalling compounds thus supporting our hypothesis (II). The results are also in accordance with the recent findings [16] on transcriptional changes of *L. bicolor* and *T. harzianum* WM24a1 in co-culture scenarios that revealed an increase in the number of differentially expressed genes (DEGs) in DC compared to MC. The authors revealed DEGs mainly assigned to metabolic pathways involved in the biosynthesis of secondary metabolites, possibly indicating the activation of secondary metabolism-based communication, especially related to α-linolenic acid metabolism and leucine and isoleucine degradation [16]. Interestingly, also the present analyses revealed that metabolites related to α-linolenic acid metabolism was decreased in abundance in WM24a1 under MC. Valine, leucine and isoleucine biosynthesis were, moreover, also decreased under both MC and DC. Together with the transcriptional analyses [16], the present metabolomic analyses serve as a first step towards understanding the complex interactions and molecular regulation behind the fungal interaction and, furthermore, support the hypothesis that fungi adjust their metabolic pathways in response to their biotic environment.

It is important to recognize that the observed accumulation or reduction of specific metabolites does not necessarily reflect transcriptional regulation of the corresponding biosynthetic pathways. These changes suggest that the pathway activity in post-translational modifications, metabolite transport, or shifts in metabolic flux might be altered [86–88]. Similar observations have been reported in fungal interaction studies, where metabolite production is triggered by physical contact or environmental cues, independent of gene expression [89, 90]. While pathway mapping based on metabolite abundance provides valuable insights into the chemical dynamics of co-cultivation, complementary transcriptomic or proteomic analyses would be required to eludicate the underlying molecular regulatory mechanisms.

### Co-culture led to varying metabolic exudates with a potential role in communication at the confrontation zone in MC

In the soil matrix as well as in *ex-situ* co-culture experiments, the diffusion capability of allelochemicals through the matrix play a crucial role in fungal interactions [85]*.* Using alluvial plots, we analysed the flow and diffusion of exudates from *L. bicolor* and *Trichoderma* mycelia until the contact zone and detected six distinct patterns of mass flow in the studied interactions. These results provide clear examples of the potential metabolic changes during *L. bicolor*—*Trichoderma* confrontation. Interesting are the changes over distance, reflected as degraded as well as novel compounds detected in the contact zone compared to PC. The specific composition of these metabolites was dependent on the interaction partner, highlighting species-specific recognition and antagonism. These metabolites have been previously associated with antifungal activity in other contexts, suggesting a possible—but unconfirmed—role in defence. In line with other present results, these analyses revealed *Trichoderma* strain-specific differences in the response of the ECM.

Some common mass features were detected from all *Trichoderma* strains; however, putative annotation was possible only for the discriminating features that mainly included oxyaromatic compounds, lipids and peptides. When *L. bicolor* was co-cultured with any of the *T. harzianum* strains, nucleotides, amino sugars and carbohydrates were detected in the interaction zone, while these compound classes were not dominant in co-culture with *T. atrobrunneum.* Interestingly, some potentially fungal defence related phenolic compounds, such as quercetin 3-O-rutinoside, salicylic acid, pyrogallin and vannilin, were identified in the interaction zone between *L. bicolor* and *Trichoderma* spp*.* These have been previously reported to inhibit the growth of *T. harzianum* [68–70] and might also be a cause of the growth inhibition we detected in the co-cultures. Altogether these diffusion patterns support the hypothesis that the studied fungi could release signaling metabolites into the growth matrix long before the hyphae can encounter each other (MC), which helps to avoid/defend a competing fungal species.

Compared to our earlier study [10], which demonstrated that *Trichoderma* species emit distinct VOCs during interactions with *L. bicolor*, the present work uncovers additional layers of chemical complexity, particularly involving soluble exudates and internal metabolite reprogramming. While Guo et al. [10] emphasized differential antagonism based on VOC emission profiles, our current findings reveal that such interactions also induce broad, strain-specific shifts in metabolic pathways, including those related to amino acid, lipid, and secondary metabolism. Particularly our spatial resolved analysis of exudate diffusion and contact-dependent metabolite accumulation offers new insights into the localized chemical dynamics within confrontation zones—an aspect never addressed in previous studies. Taken together, this comprehensive comparison highlights the roles of both volatile and non-volatile metabolites in mediating fungal recognition and competitive interactions.

## Conclusions

In this study, we combined putatively annotated volatile and soluble metabolomic data to investigate the chemical processes underlying interactions between plant-beneficial fungi that might exhibit antagonistic behaviour under in vitro conditions. Notwithstanding the restricted power of in vitro co-cultures in the laboratory and the limited annotation of metabolites, our study offers compelling evidence for a high degree of metabolic diversity in the interacting fungi grown under similar growth conditions as pure and co-cultures. The observed metabolite shifts suggest a form of chemical responsiveness, which may indicate perception of non-self-signals; however, direct evidence for recognition mechanisms remains to be established. It is important to note that our study cannot be considered as a replacement for field studies conducted under natural conditions, where complex microbial consortium is present. Nevertheless, it offers a preliminary basis for further investigation of analogous interactions under more natural conditions, and for conducting three-partite interactions involving a common plant host. Incorporating transcriptomics, proteomics, and functional assays into future research will be necessary to determine how these metabolic signals integrate into broader recognition and defence pathways in natural settings. Such studies are essential to understand how fungal–fungal interactions influence, and are influenced by, plant signalling networks in the shared rhizosphere. In conclusion, the results of this study provide insight into the potential of volatile and soluble allelochemicals in mediating interspecific communication, recognition and competition between *Trichoderma* and an ECM (*L. bicolor*).

## Supplementary Information

Supplementary material 1. Supplementary Methods.

Supplementary material 2. Supplementary Figures and Tables.

## Data Availability

The supplementary filesets generated and/or analysed during the current study are available in the open science framework data repository and can be accessed following the link: https://osf.io/c357a/?view_only=7397fa80cc9846869879168112478139.

## Abbreviations

*Th*: *Trichoderma harzianum*
*T.arb*: *Trichoderma atrobrunneum*
*Lb*: *Laccaria bicolor*
AC: Aerial contact
MC: Media contact
DC: Direct contact
VOC: Volatile organic compound
PCA: Principal component analysis
OPLS-DA: Orthogonal partial least square regression -discriminant analysis
HCA: Hierarchical clustering analysis
