# Supplementary material for: Strain and contact-dependent metabolomic reprogramming reveals distinct interaction strategies between Laccaria bicolor and Trichoderma
Source: Fungal Biol Biotechnol. 2025 Jul 22;12:13. doi: 10.1186/s40694-025-00204-w (PMC12285104; doi:10.1186/s40694-025-00204-w)
Supplement: Supplementary file 1 — Supplementary material 1. Supplementary Methods. [file 40694_2025_204_MOESM1_ESM.docx]

**Supplementary Methods**

**Deciphering plant-beneficial fungal interactions: Unravelling metabolic diversity that underpins communication between *Laccaria bicolor* and *Trichoderma***

**Prasath Balaji Sivaprakasam Padmanaban^1^, Pia Stange^2^, Baris Weber^1^, Andrea Ghirardo^1^, Karin Pritsch^1^, Tanja Karl^2^, J. Philipp Benz^2^, Maaria Rosenkranz^1,3*^, Jörg-Peter Schnitzler^1^,**

^1^Research Unit Environmental Simulation (EUS), Helmholtz Munich, Neuherberg, Germany

^2^Professorship Fungal Biotechnology in Wood Science, Wood Research Munich, TUM School of Life Sciences, Technical University of Munich, Freising, Germany

^3^Institute of Plant Sciences, Ecology and Conservation Biology, University of Regensburg, Germany

***Correspondence:**Corresponding Author:

Maaria Rosenkranz

[maaria.rosenkranz@biologie.uni-regensburg.de](mailto:maaria.rosenkranz@biologie.uni-regensburg.de)

**Method S1**. **Composition of the Modified MMN-Medium (**[**1**](#_ENREF_1)**) used in the experiment:**

| **Substrate** | **Company** | **Concentration/ L** |
| --- | --- | --- |
| Glucose | Duchefa | **10 g** |
| Maltose | Duchefa | **3 g** |
| NH_4_-Tartrat | VWR | 2,5 g |
| (NH_4_)_2_SO_4_ | Merck | 0,25 g |
| KH_2_PO_4_ | Duchefa | 0,5 |
| MgSO_4_ 7 H_2_O | Duchefa | 0,15 |
| CaCl_2_ 2 H_2_O | Merck | 0,05 |
| NaCl | Merck | 0,025 g |
| Thiamin HCl * 0,1%ig  (10 mg/10 ml water, sterile Filtrate) | Merck | **100 µl** |
| FeCl_3_ * 1%ig  (1 g/100 ml Water, sterile Filtrate) | Merck | **1 ml** |
| Gelrite | Duchefa | **1 %** |

To prepare the Modified MMN-Medium pH is set to 5.2, the medium is autoclaved and, after cooling down to ca. 60°C Thiamin-HCl and FeCl_3_ are added.

**Method S2**. **Primer pairs used for fungal identification (**[**2**](#_ENREF_2)**):**

| **Target** | **Primer sequence (5’-3’)** | **Reference** |
| --- | --- | --- |
| **ITS 1** | TCCGTAGGTGAACCTGCGG | ([3](#_ENREF_3)) |
| **ITS 4** | TCCTCCGCTTATTGATATGC |  |
| **EF1** | ATGGGTAAGGARGACAAGAC | ([4](#_ENREF_4)) |
| **EF2** | GGARGTACCAGTSATCATGTT |  |
| **fRPB2-5f** | GAYGAYMGWGATCAYTTYGG | ([5](#_ENREF_5)) |
| **fRPB2-7cr** | CCCATRGCTTGTYYRCCCAT |  |

**Method S3: Detailed extraction protocols (A) and parameters (B) of the methods used for non-targeted metabolomics using an ultra-performance liquid chromatography (UPLC) system coupled with ultra-high resolution (UHR) tandem quadrupole/time-of-flight (QqToF) mass spectrometry (MS):**

1. For the extraction process, 800 µL of a cold (5°C) mixture of methanol: 2-propanol: water (in a ratio 1:1:1, v/v/v) extraction solvent was added to 25 mg of mycelium and freeze-dried media. This solvent contained 50 µL of an internal standard (IS) mixture (Supplementary Methods Table S3). The samples were gently mixed in a 2 mL polypropylene tube for 1 minute. These mixtures were then ultrasonicated for 10 min in a water bath held at approximately 5°C. After sonication, the solution was centrifuged at 10g for 10 minutes at 4°C. This step resulted in the collection of 640 µL of supernatant. The collected supernatant was then subjected to drying using a SpeedVac system (Univapo 150H, Uniequip, Planegg, Germany). The resulting residue was reconstituted in 400 µL of 50% (v/v) acetonitrile in water. After a mixing step of 1 min and subsequent centrifugation at 10 g for 10 min at 5°C, 300 µL of supernatant was carefully transferred into 350 µL amber glass vials (following ([6](#_ENREF_6))). The instrumentation comprises an Ultimate 3000RS UPLC system (Thermo Fisher, Bremen, Germany), a Bruker Impact II QqToF mass spectrometer, and an Apollo II electrospray ionisation (ESI) source (Bruker Daltonic, Bremen, Germany) ([6](#_ENREF_6)).
2. The following solvents (Sigma-Aldrich, Taufkirchen, Germany) were used for elution: solvent A (Water (Lichrosolv®) with 99.9% (v/v) Formic acid (Lichropur®) and solvent B (Acetonitrile (Honeywell®) with 99.9% (v/v) Formic acid (Lichropur®)).

**The gradient program for reverse-phase liquid chromatography (RPLC) using an Acquity UPLC® BEH C18 (1.7µm) column (Water Cooperation, Eschborn, Germany) with a dimension of 2.1x 150 mm was as follows:**

- 0-1 min: 95% A (isocratic),

- 1-15 min: 95-70% A,

- 15-17 min: 70-20% A,

- 17-20 min: 20% A (isocratic),

- 20-22 min: 20-0.5% A,

- 22-27 min: 0.5% A (isocratic),

- 27-29 min: 0.5-95% A,

- 29-31 min: 95% A (isocratic).

**For hydrophilic interaction liquid chromatography (HILIC) separation using an Acquity UPLC® BEH AMIDE column (1.7µm) (Water Cooperation) with a dimension of 2.1x 100 mm, the gradient applied was:**

- 0-1 min: 5% A (isocratic),

- 1-16 min: 5-30% A,

- 16-18 min: 30-80% A,

- 18-19 min: 80% A (isocratic),

- 19-20 min: 80-95% A,

- 20-21 min: 95-4.5% A.

In both methods, the flow rate was maintained at 0.4 mL/min, with the column temperature set at 40°C throughout the separation process. The injection volume was 5 µL.

Mass calibration was performed using a mixture containing 50 mL water, 50 mL 2-propanol, 1 mL NaOH, and 200 µL formic acid.

**The mass spectrometer (MS) operated in both positive (+) and negative (-) ionization modes, with the following parameters:**

- Nebulizer pressure: 2.0 bar,

- Dry gas flow: 8.0 L min^-1^,

- Dry gas temperature: 200°C,

- Capillary voltage: 4500 V for (+) and 3500 V for (-),

- Endplate offset: 500 V,

- Mass range: 20–2000 m/z.

For fragmentation studies and metabolite identification, tandem mass spectrometry (MS/MS) was performed using the auto MS/MS acquisition mode, with the smart exclusion for precursor selection. A precursor was selected for MS/MS when the difference between the rolling average of the set number of spectra and the actual spectra exceeded an absolute threshold of 1000 counts. Collision energies ranged from 5 to 20 eV for ion fragmentation in the collision cell.

**Method S4. LC-MS data processing parameters in Metaboscape 4.0® (Bruker Daltonics):**

| Peak detection | |  |
| --- | --- | --- |
| Intensity threshold/spectra | | 1000 |
| Minimum peak length /spectra | | 7 |
| Feature signal | | Area |
| Recursive Feature extraction | |  |
| Minimum peak length recursive/spectra | | 6 |
| Minimum number of features for extraction | | 2/5 |
| Presence of features in minimum number of analyses | | 2/5 |
| MS/MS import method | | Average |
| EIC correlation | | 0.7 |
| Ionization mode | **Positive mode** | **Negative mode** |
| Primary ion | [M+H]^+^ | [M-H]^-^ |
| Seed ions | [M+Na]^+^,[M+K]^+^ | [M+Cl]^-^ |
| Common ions | [M-H20+H]^+^,  [2M+H]^+^,  [M+H+CH3CN]^+^ | [M-H20+H]^-^,  [2M+H]^-^,  [M-H+CH3CN]^-^  [M-H+HCOOH]^-^ |

**Method S5**. **List of internal standards (Sigma-Aldrich) used for normalisation:**

| Internal standard | Neutral Mass | Mode |
| --- | --- | --- |
| Bergapten | 216.04 | + |
| Plumbagin | 188.05 | + |
| Dihydrocaffeic acid | 182.06 | - |
| 3,4-dihydromandelic acid | 184.04 | - |

**References:**

1. Müller A, Faubert P, Hagen M, zu Castell W, Polle A, Schnitzler J-P, et al. Volatile profiles of fungi – Chemotyping of species and ecological functions. Fungal Genetics and Biology. 2013;54:25-33.

2. Stange P, Seidl S, Karl T, Benz JP. Evaluation of Trichoderma isolates as biocontrol measure against Claviceps purpurea. European Journal of Plant Pathology. 2023;167(4):651-75.

3. White TJ, Bruns T, Lee S, Taylor J. 38 - AMPLIFICATION AND DIRECT SEQUENCING OF FUNGAL RIBOSOMAL RNA GENES FOR PHYLOGENETICS. In: Innis MA, Gelfand DH, Sninsky JJ, White TJ, editors. PCR Protocols. San Diego: Academic Press; 1990. p. 315-22.

4. O’Donnell K, Kistler HC, Cigelnik E, Ploetz RC. Multiple evolutionary origins of the fungus causing Panama disease of banana: Concordant evidence from nuclear and mitochondrial gene genealogies. Proceedings of the National Academy of Sciences. 1998;95(5):2044-9.

5. Liu YJ, Whelen S, Hall BD. Phylogenetic relationships among ascomycetes: evidence from an RNA polymerse II subunit. Molecular Biology and Evolution. 1999;16(12):1799-808.

6. Bertić M, Schroeder H, Kersten B, Fladung M, Orgel F, Buegger F, et al. European oak chemical diversity – from ecotypes to herbivore resistance. New Phytologist. 2021;232(2):818-34.
