# Supplementary material for: Strain and contact-dependent metabolomic reprogramming reveals distinct interaction strategies between Laccaria bicolor and Trichoderma
Source: Fungal Biol Biotechnol. 2025 Jul 22;12:13. doi: 10.1186/s40694-025-00204-w (PMC12285104; doi:10.1186/s40694-025-00204-w)
Supplement: Supplementary file 2 — Supplementary material 2. Supplementary Figures and Tables. [file 40694_2025_204_MOESM2_ESM.docx]

**Supplementary Figures and Tables**

**Deciphering plant-beneficial fungal interactions: Unravelling metabolic diversity that underpins communication between *Laccaria bicolor* and *Trichoderma***

**Prasath Balaji Sivaprakasam Padmanaban^1^, Pia Stange^2^, Baris Weber^1^, Andrea Ghirardo^1^, Karin Pritsch^1^, Tanja Karl^2^, J. Philipp Benz^2^, Maaria Rosenkranz^1,3*^, Jörg-Peter Schnitzler^1^,**

^1^Research Unit Environmental Simulation (EUS), Helmholtz Munich, Neuherberg, Germany

^2^Professorship Fungal Biotechnology in Wood Science, Wood Research Munich, TUM School of Life Sciences, Technical University of Munich, Freising, Germany

^3^Institute of Plant Sciences, Ecology and Conservation Biology, University of Regensburg, Germany

***Correspondence:**Corresponding Author:

Maaria Rosenkranz

[maaria.rosenkranz@biologie.uni-regensburg.de](mailto:maaria.rosenkranz@biologie.uni-regensburg.de)

**Supplementary Figures:**


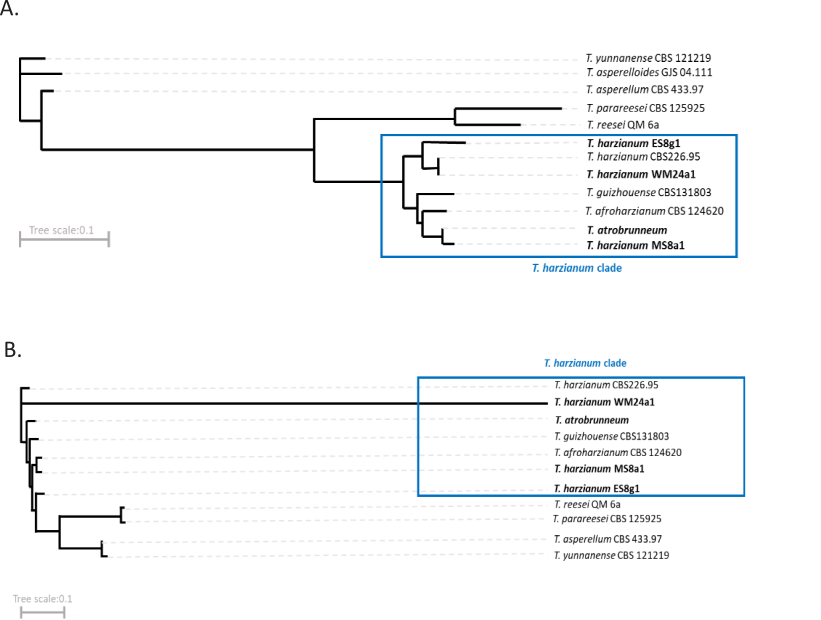


**Fig S1.** The phylogenetic tree of *Trichoderma* spp., included in the study (in bold) with other reference *Trichoderma* species ([1](#_ENREF_1)) was constructed from sequences of the *translation elongation factor 1α* (*tef1*) (A) and *RNA polymerase II subunit B* (*rpb2*) (B) genes, representing relationships within the genus *Trichoderma.*


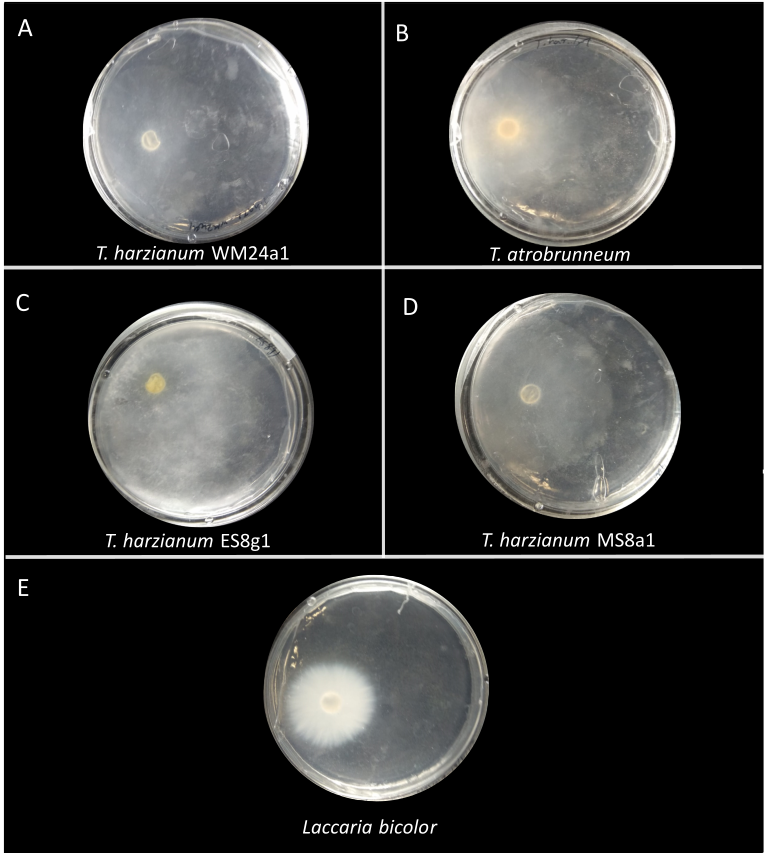


**Fig S2.** Representative images of pure cultures of the different *Trichoderma* strains used for co-cultivation on day 3. Pure culture of (A)*Trichoderma harzianum* (WM24a1), (B) *Trichoderma atrobrunneum,* (C) *Trichoderma harzianum* (ES8g1), (D) *Trichoderma harzianum* (MS8a1) and (E) pure culture of *Laccaria bicolor* (strain S238N) on day 17.


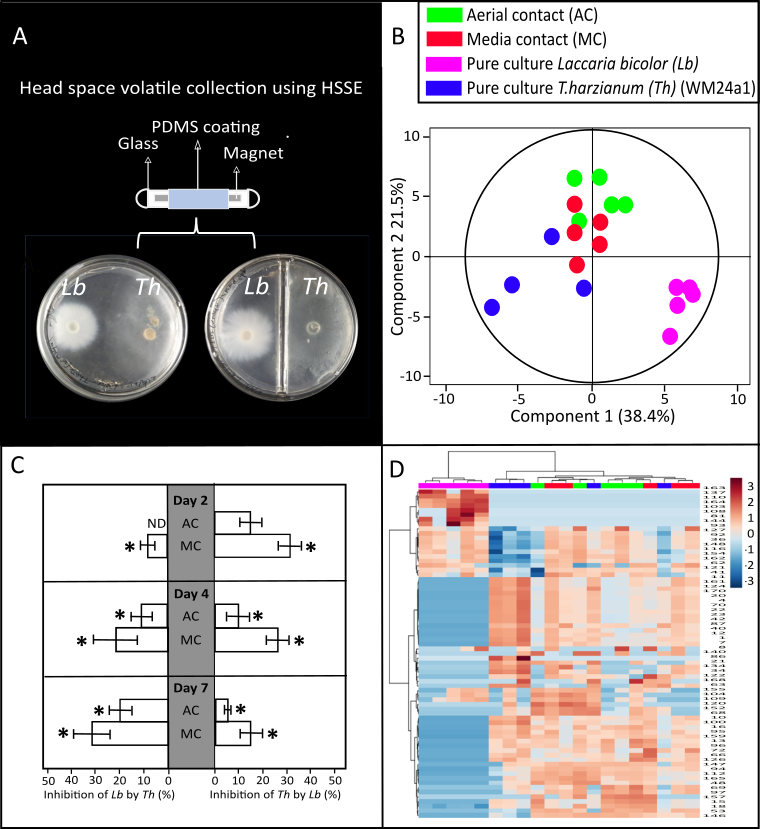
**Fig S3.** VOC analysis of of *Laccaria bicolor (Lb)* and *T. harzianum (Th) (*WM24a1*) (Th)* co-cultivated either in aerial contact (AC) or media contact (MC). (A) Experimental setup of the VOC collection from the fungal AC and DC co-cultivation in split and non-split petri plates by twisters employing Headspace sorptive extraction (HSSE) technique. (B) orthogonal partial least square regression discriminant analysis (OPLS-DA) showing differences among VOC profiles under different levels of co-cultivation, (C) Growth inhibition of *Th* on *Lb* (left) and *Lb* on *Th* (right) under different levels of co-cultivation compared to growth on pure cultures. ND: not detected. Significances within each day are denoted as asterisks (one-way ANOVA and Tukey HSD, p < 0.05); mean ± SE; Values are average of 5 replicates and (D) Hierarchical clustering analysis of the volatile concentration of the VOCs from the two fungi grown as MC, AC or pure culture*.*


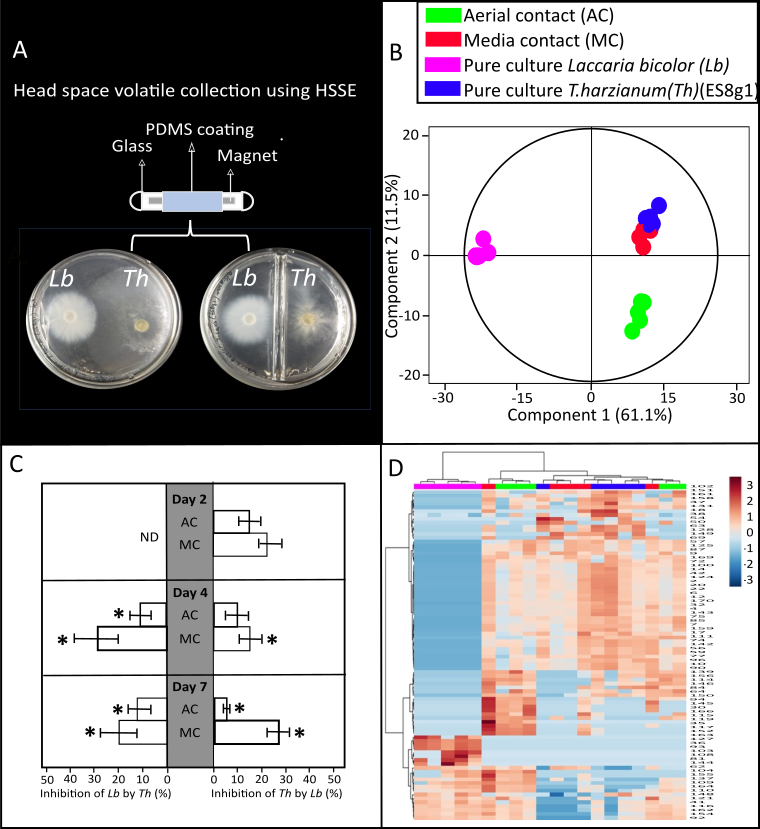
 **Fig S4.** VOC analysis of of *Laccaria bicolor (Lb)* and *T. harzianum (Th) (*ES8g1*) (Th)* co-cultivated either in aerial contact (AC) or media contact (MC). (A) Experimental setup of the VOC collection from the fungal AC and DC co-cultivation in split and non-split petri plates by twisters employing Headspace sorptive extraction (HSSE) technique. (B) orthogonal partial least square regression discriminant analysis (OPLS-DA) showing differences among VOC profiles under different levels of co-cultivation, (C) Growth inhibition of *Th* on *Lb* (left) and *Lb* on *Th* (right) under different levels of co-cultivation compared to growth on pure cultures. ND: not detected. Significances within each day are denoted as asterisks (one-way ANOVA and Tukey HSD, p < 0.05); mean ± SE; Values are average of 5 replicates and (D) Hierarchical clustering analysis of the volatile concentration of the VOCs from the two fungi grown as MC, AC or pure culture*.*


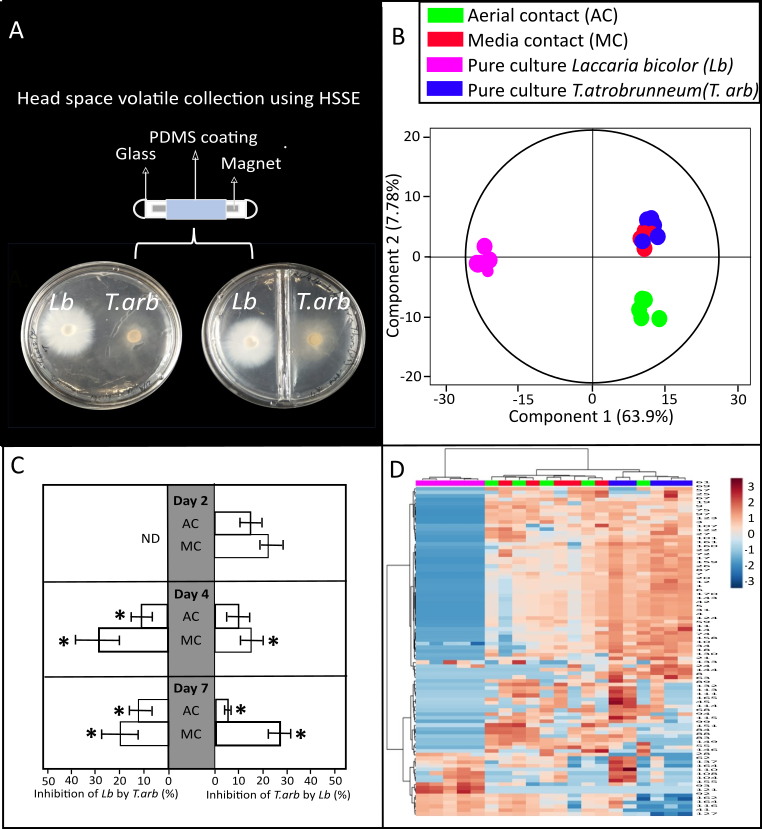
**Fig S5.** VOC analysis of of *Laccaria bicolor* (Lb) and *T. atrobrunneum (T.arb*) co-cultivated either in aerial contact (AC) or media contact (MC). (A) Experimental setup of the VOC collection from the fungal AC and DC co-cultivation in split and non-split petri plates by twisters employing Headspace sorptive extraction (HSSE) technique. (B) orthogonal partial least square regression discriminant analysis (OPLS-DA) showing differences among VOC profiles under different levels of co-cultivation, (C) Growth inhibition of *T.arb* on *Lb* (left) and *Lb* on *T.arb* (right) under different levels of co-cultivation compared to growth on pure cultures. ND: not detected. Significances within each day are denoted as asterisks (one-way ANOVA and Tukey HSD, p < 0.05); mean ± SE; Values are average of 5 replicates and (D) Hierarchical clustering analysis of the volatile concentration of the VOCs from the two fungi grown as MC, AC or pure culture.


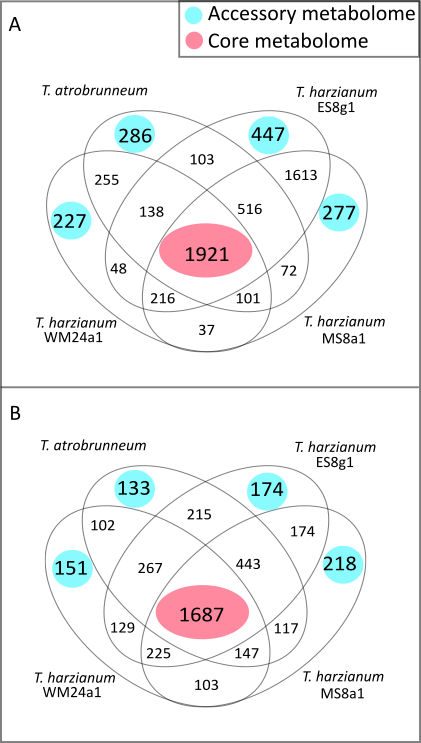
**Fig S6.** Accessory (unique) and core (common) metabolome in the hyphae (A) and media (B) of pure cultures in different *Trichoderma* species.

**Fig S7.** Metabolomic analysis of the hyphae from the co-cultivation experiment of *Laccaria bicolor* (*Lb*) and *T. harzianum (Th) (*WM24a1*).* (A) Exemplary image of the confrontation assay showing the three different zones of sampling of hyphae and
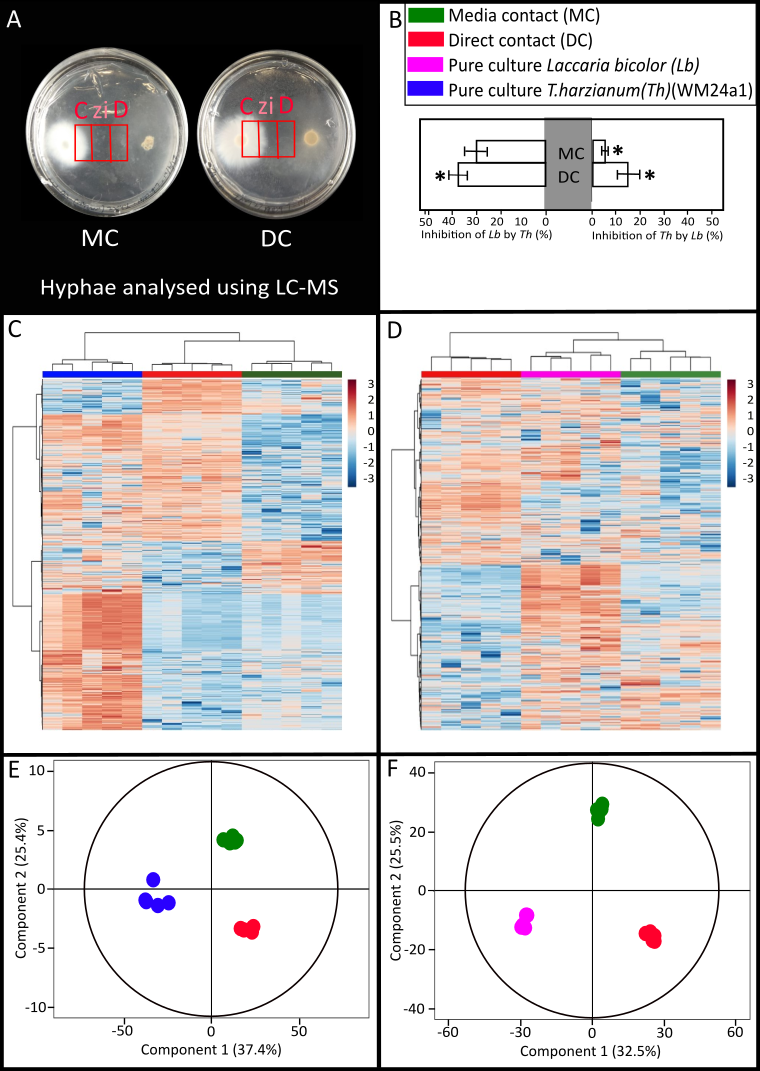
media across media contact (MC) and direct contact (DC). (B) Growth inhibition of *(Th)* on *Lb* (left) and vice versa (right) under different levels of co-cultivation compared to pure cultures. Significances are denoted as asterisks (one-way ANOVA and Tukey HSD, p < 0.05); mean ± SE; Values are average of 5 replicates. (C, D) Hierarchical clustering analysis of the peak area of features from cultures of (C) *Lb* and (D) *Th* hyphae grown in media contact (MC), direct contact (DC) and pure cultures. Features are selected based on having a VIP (Variable Importance of Projection) score >1 and regression coefficients to compute HCA. (E, F) Orthogonal partial least square regression discriminant analysis (OPLS-DA) showing differences among metabolic features under different levels of co-cultivation in (E) *Lb* & in (F) *Th.*


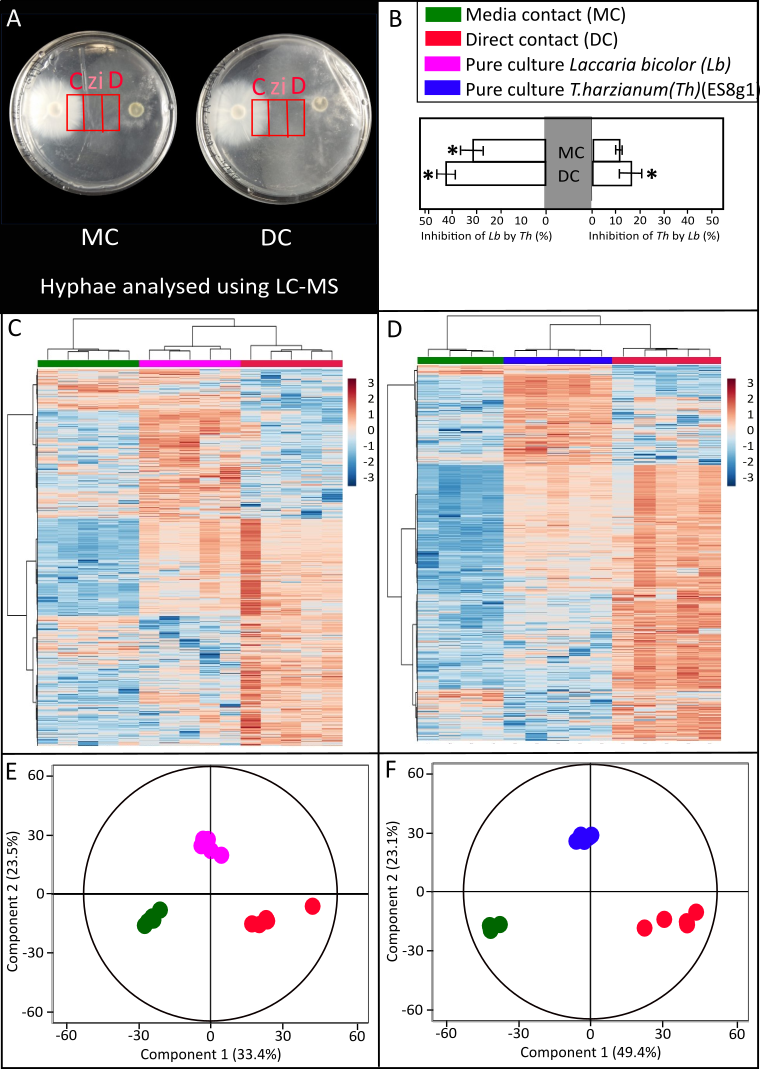
**Fig S8.** Metabolomic analysis of the hyphae from the co-cultivation experiment of *Laccaria bicolor* (*Lb*) and *T. harzianum (Th) (*ES8g1*).* (A) Exemplary image of the confrontation assay showing the three different zones of sampling of hyphae and media across media contact (MC) and direct contact (DC). (B) Growth inhibition of *Th* on *Lb* (left) and vice versa (right) under different levels of co-cultivation compared to pure cultures. Significances are denoted as asterisks (one-way ANOVA and Tukey HSD, p < 0.05); mean ± SE; Values are average of 5 replicates. (C, D) Hierarchical clustering analysis of the peak area of features from cultures of (C) *Laccaria* and (D) *Th* hyphae grown in media contact (MC), direct contact (DC) and pure cultures. Features are selected based on having a VIP (Variable Importance of Projection) score >1 and regression coefficients to compute HCA. (E, F) Orthogonal partial least square regression discriminant analysis (OPLS-DA) showing differences among metabolic features under different levels of co-cultivation in (E) *Lb* & in (F) *Th*.


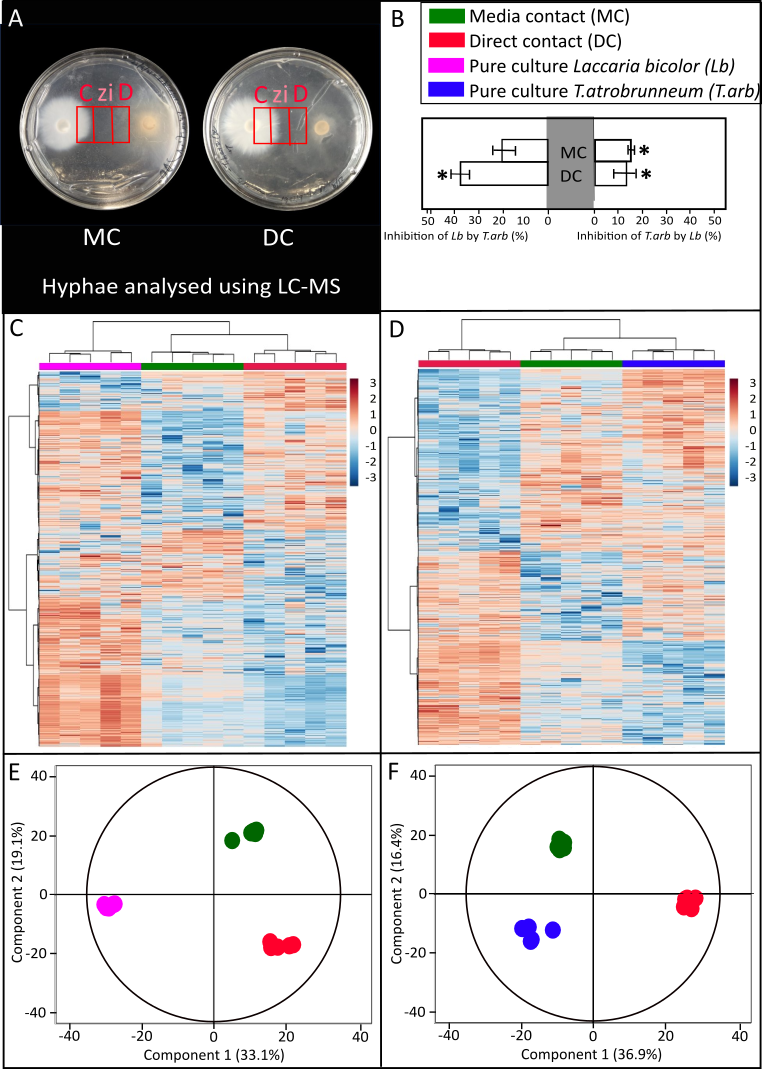
**Fig S9.** Metabolomic analysis of the hyphae from the co-cultivation experiment of *Laccaria bicolor* (*Lb*) and *T. atrobrunneum (T.arb).* (A) Exemplary image of the confrontation assay showing the three different zones of sampling of hyphae and media across media contact (MC) and direct contact (DC). (B) Growth inhibition of *T. arb* on *Lb* (left) and vice versa (right) under different levels of co-cultivation compared to pure cultures. Significances are denoted as asterisks (one-way ANOVA and Tukey HSD, p < 0.05); mean ± SE; Values are average of 5 replicates. (C, D) Hierarchical clustering analysis of the peak area of features from cultures of (C) *Laccaria* and (D) *T. arb* hyphae grown in media contact (MC) direct contact (DC) and pure cultures. Features are selected based on having a VIP (Variable Importance of Projection) score >1 and regression coefficients to compute HCA. (E, F) Orthogonal partial least square regression discriminant analysis (OPLS-DA) showing differences among metabolic features under different levels of co-cultivation in (E) *Lb* & in (F) *T. arb.*


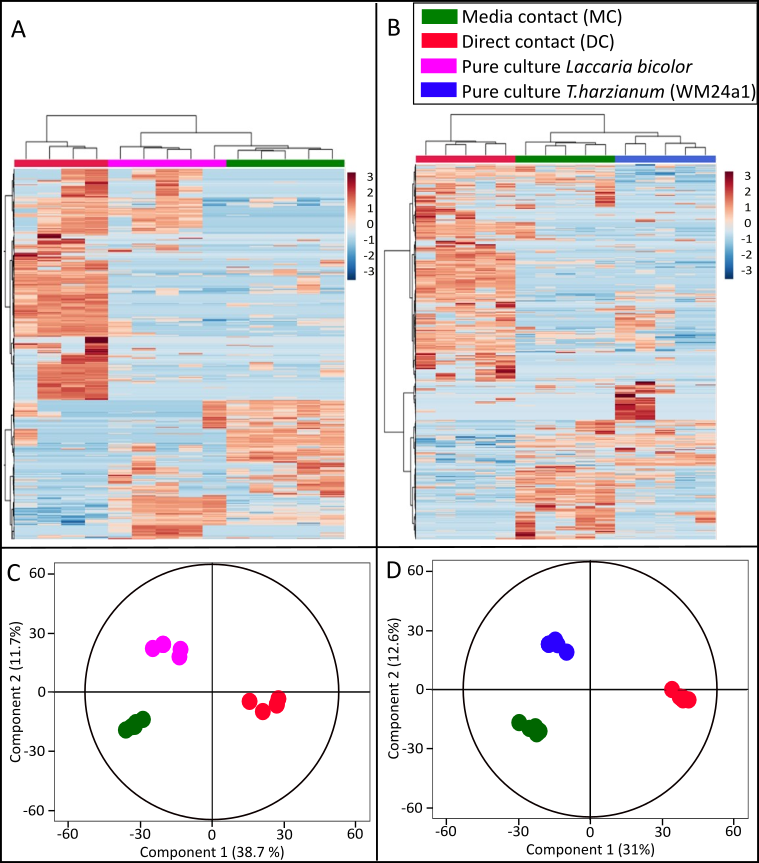
**Fig S10.** Metabolomic analysis of the media from the co-cultivation experiment of *Laccaria bicolor* (*Lb*) and *T. harzianum (*WM24a1*) (Th).* (A, B) Hierarchical clustering analysis of the peak area of features from cultures of (A) *Lb* and (B) *Th* grown as media contact (MC), direct contact (DC) and pure cultures. (C, D) Orthogonal partial least square regression discriminant analysis (OPLS-DA) showing differences among metabolic features in media under different levels of co-cultivation in (C) *Lb* & (D)*Th*.


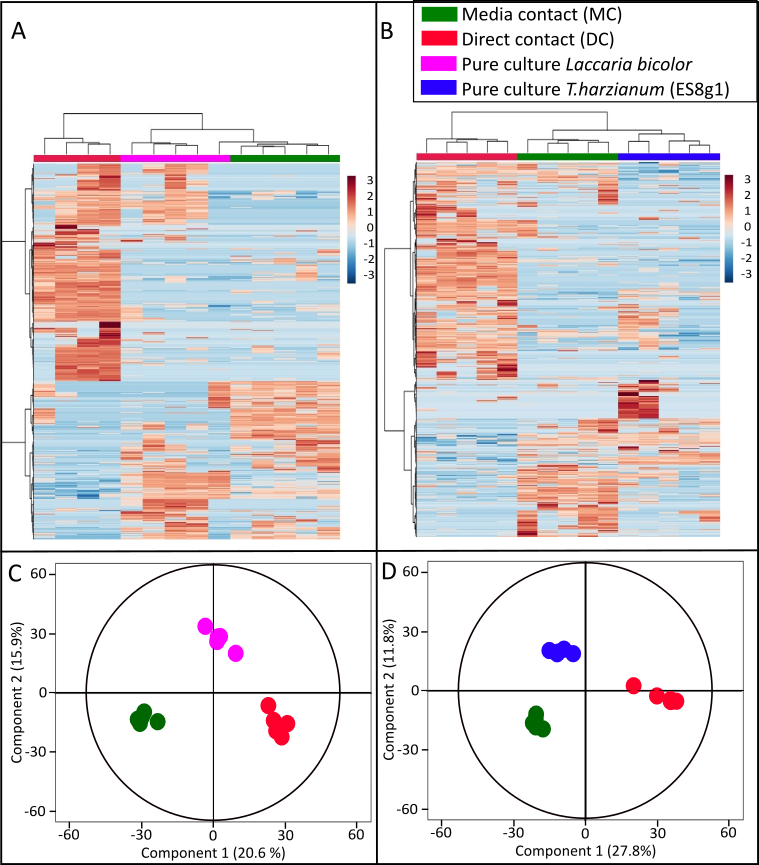
 **Fig S11.** Metabolomic analysis of the media from the co-cultivation experiment of *Laccaria bicolor* (*Lb*) and *T. harzianum (*ES8g1*) (Th).* (A, B) Hierarchical clustering analysis of the peak area of features from cultures of (A) *Lb* and (B) *Th* grown also as media contact (MC) direct contact (DC) and pure cultures. (C, D) Orthogonal partial least square regression discriminant analysis (OPLS-DA) showing differences among metabolic features in media under different levels of co-cultivation in (C) *Lb* & (D)*Th*.


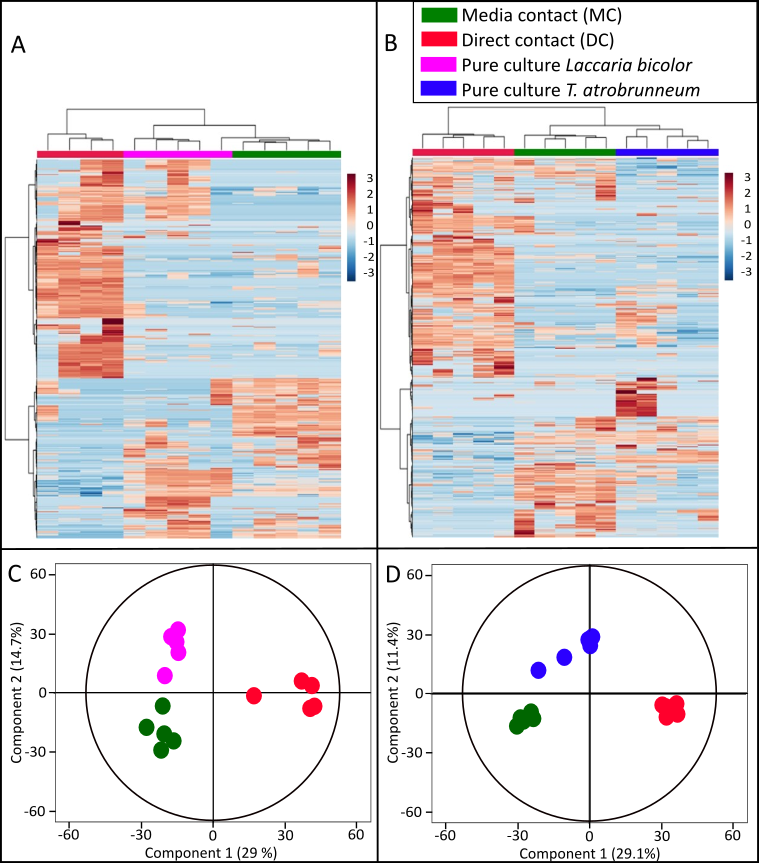


**Fig S12.** Metabolomic analysis of the media from the co-cultivation experiment of *Laccaria bicolor* (*Lb*) and *T. atrobrunneum (T.arb).* (A, B) Hierarchical clustering analysis of the peak area of features from cultures of (A) *Lb* and (B) *Tarb* grown also as media contact (MC) direct contact (DC) and pure cultures. (C, D) Orthogonal partial least square regression discriminant analysis (OPLS-DA) showing differences among metabolic features in media under different levels of co-cultivation in (C) *Lb* & (D)*Tarb*.


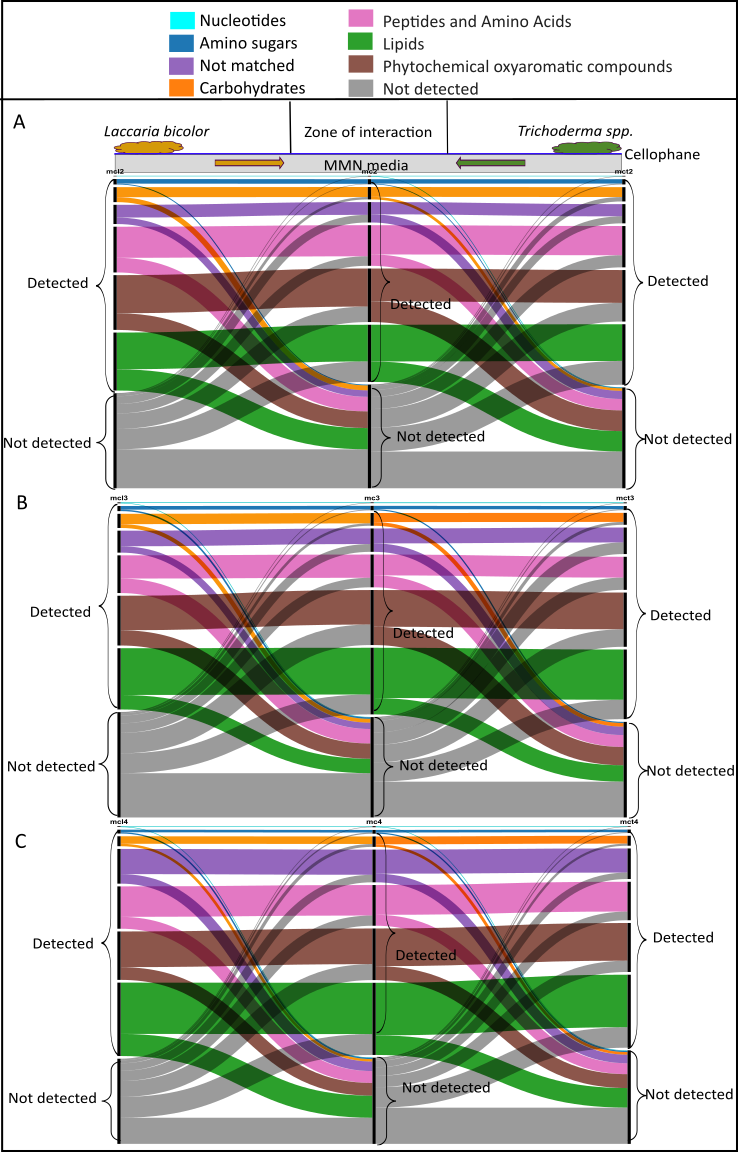


**Fig S13.** Alluvial plot showing the flow of exudates from *Laccaria bicolor* to (A)*T. harzianum (*WM24a1*)*, (B) *T. harzianum (*ES8g1*)*, (C) *T. atrobrunneum* and vice versa through the common growth media in media contact of the co-cultivation.

**Supplementary Tables:**

**Table S1. Total emission strength of the different terpene classes across *Trichoderma* strains (in pmol cm^2^ hour^-1^):**

| CLASS | WM24a1 | WM24a1_AC | WM24a1_MC |
| --- | --- | --- | --- |
| Monoterpenes | 0 | 0 | 0 |
| Oxygenated monoterpenes | 0.28 | 5.07 | 2.18 |
| Sesquiterpenes | 104.26 | 109.86 | 81.68 |
| Oxygenated sesquiterpenes | 3.88 | 4.44 | 2.85 |
|  | **ES8g1** | **ES8g1_AC** | **ES8g1_MC** |
| Monoterpenes | 0.07 | 3.12 | 2.62 |
| Oxygenated monoterpenes | 0.19 | 0.30 | 0.15 |
| Sesquiterpenes | 95.04 | 52.59 | 76.40 |
| Oxygenated sesquiterpenes | 3.21 | 1.54 | 2.29 |
|  | **MS8a1** | **MS8a1_AC** | **MS8a1_MC** |
| Monoterpenes | 14.14 | 7.11 | 9.91 |
| Oxygenated monoterpenes | 0 | 0 | 0 |
| Sesquiterpenes | 23.37 | 6.26 | 10.03 |
| Oxygenated sesquiterpenes | 0.63 | 0.44 | 0.63 |
|  | ***T.arb*** | ***T.arb_AC*** | ***T.arb_MC*** |
| Monoterpenes | 21.74 | 34.29 | 7.46 |
| Oxygenated monoterpenes | 0.48 | 0.63 | 0.51 |
| Sesquiterpenes | 131.98 | 56.07 | 49.19 |
| Oxygenated sesquiterpenes | 3.62 | 1.56 | 1.35 |
|  | ***L. bicolor*** |  |  |
| Monoterpenes | 13.41 |  |  |
| Oxygenated monoterpenes | 0 |  |  |
| Sesquiterpenes | 0.48 |  |  |
| Oxygenated sesquiterpenes | 0 |  |  |

**Table S2. Differentially regulated metabolic features in hyphae of the fungal strains under different co-cultivation scenarios:**

| *Trichoderma* strains on contact with *Laccaria* | | | | | | | | |
| --- | --- | --- | --- | --- | --- | --- | --- | --- |
|  | **WM24a1** | | **ES8g1** | | **MS8a1** | | ***T. atrobrunneum*** | |
|  | **Up** | **Down** | **Up** | **Down** | **Up** | **Down** | **Up** | **Down** |
| MC | 60 | 314 | 114 | 511 | 5 | 488 | 135 | 79 |
| DC | 162 | 300 | 595 | 522 | 159 | 319 | 434 | 332 |
| *Laccaria* on contact with *Trichoderma* strains | | | | | | | | |
| MC | 155 | 444 | 71 | 409 | 22 | 401 | 430 | 103 |
| DC | 126 | 339 | 102 | 235 | 196 | 254 | 110 | 395 |

**Table S3. Differentially regulated metabolic features in the exudates of the fungal strains under different co-cultivation scenarios:**

| *Trichoderma* strains on contact with *Laccaria* | | | | | | | | |
| --- | --- | --- | --- | --- | --- | --- | --- | --- |
|  | **WM24a1** | | **ES8g1** | | **MS8a1** | | ***T. atrobrunneum*** | |
|  | **Up** | **Down** | **Up** | **Down** | **Up** | **Down** | **Up** | **Down** |
| MC | 25 | 14 | 3 | 60 | 22 | 70 | 3 | 11 |
| DC | 16 | 26 | 11 | 19 | 47 | 112 | 4 | 6 |
| *Laccaria* on contact with *Trichoderma* strains | | | | | | | | |
| MC | 31 | 74 | 49 | 29 | 26 | 15 | 9 | 50 |
| DC | 16 | 77 | 12 | 89 | 19 | 59 | 20 | 76 |

**References:**

1. Cai F, Druzhinina IS. In honor of John Bissett: authoritative guidelines on molecular identification of Trichoderma. Fungal Diversity. 2021;107(1):1-69.
